# The Aegean in the Early 7th Millennium BC: Maritime Networks and Colonization

**DOI:** 10.1007/s10963-015-9090-8

**Published:** 2015-12-10

**Authors:** B. Horejs, B. Milić, F. Ostmann, U. Thanheiser, B. Weninger, A. Galik

**Affiliations:** Institute for Oriental and European Archaeology (OREA), Austrian Academy of Sciences, Fleischmarkt 20-22, 1010 Vienna, Austria; Institute for Anatomy, Histology and Embryology, Veterinary Medicine University Vienna, Veterinärplatz 1, 1210 Vienna, Austria; Vienna Institute for Archaeological Science (VIAS), University of Vienna, Althanstrasse 14, 1090 Vienna, Austria; Institut für Ur- und Frühgeschichte, University of Cologne, Albertus-Magnus-Platz, 50931 Cologne, Germany; Department of Prehistory, Istanbul University, 34134 Laleli, Istanbul Turkey

**Keywords:** Neolithization, Western Anatolia, Aegean, Maritime networks and colonization, Çukuriçi Höyük

## Abstract

The process of Near Eastern neolithization and its westward expansion from the core zone in the Levant and upper Mesopotamia has been broadly discussed in recent decades, and many models have been developed to describe the spread of early farming in terms of its timing, structure, geography and sociocultural impact. Until now, based on recent intensive investigations in northwestern and western Anatolia, the discussion has mainly centred on the importance of Anatolian inland routes for the westward spread of neolithization. This contribution focuses on the potential impact of east Mediterranean and Aegean maritime networks on the spread of the Neolithic lifestyle to the western edge of the Anatolian subcontinent in the earliest phases of sedentism. Employing the *longue durée* model and the concept of ‘social memory’, we will discuss the arrival of new groups via established maritime routes. The existence of maritime networks prior to the spread of farming is already indicated by the high mobility of Epipalaeolithic/Mesolithic groups exploring the Aegean and east Mediterranean seas, and reaching, for example, the Cyclades and Cyprus. Successful navigation by these early mobile groups across the open sea is attested by the distribution of Melian obsidian. The potential existence of an additional Pre-Pottery Neolithic (PPN) obsidian network that operated between Cappadocia/Cilicia and Cyprus further hints at the importance of maritime coastal trade. Since both the coastal and the high seas networks were apparently already well established in this early period, we may further assume appropriate knowledge of geographic routes, navigational technology and other aspects of successful seafaring. This Mesolithic/PPN maritime know-how package appears to have been used by later groups, in the early 7th millennium calBC, exploring the centre of the Anatolian Aegean coast, and in time establishing some of the first permanent settlements in that region. In the present paper, we link this background of newcomers to the western edge of Anatolia with new excavation results from Çukuriçi Höyük, which we have analysed in terms of subsistence strategies, materiality, technology and symbolism. Additionally, further detailed studies of nutrition and obsidian procurement shed light on the distinct maritime affinity of the early settlers in our case study, something that, in our view, can hardly be attributed to inland farming societies. We propose a maritime colonization in the 7th millennium via routes from the eastern Mediterranean to the eastern Aegean, based on previously developed sea networks. The pronounced maritime affinity of these farming and herding societies allows us to identify traces of earlier PPN concepts still embedded in the social-cultural memories of the newcomers and incorporated in a new local and regional Neolithic identity.

## Introduction

The process of neolithization has been a hot topic in archaeology ever since V. G. Childe (1957), and has never lost its appeal to researchers. However, even today perhaps the most striking characteristic of this research field is not its scientific content *per se* but its quite remarkable publication output. This output is characterized by the most diverse of all possible interdisciplinary research, from archaeology, language, DNA, isotopic and spectrographic analysis—all at the same time, although not necessarily in parallel. One reason for the persistent interest in the spread of farming may be the enduring possibility of attaining some fundamental advance in our understanding of the human mind. This is especially warranted in areas linked to the Fertile Crescent, one of the world’s core zones of neolithization.

The archaeological data of this core zone have been used to develop a wide variety of potential Neolithic trajectories, most often established as cultural lines simply running from east to west. Despite the cultural impact of such an assumed ‘international’ process (which is indeed obvious for our study region, the eastern Aegean, at least on longer time-scales), all that such trajectory models ultimately achieve is to illustrate the one actual outcome of an otherwise multi-faceted neolithization process. Within a global context, the adoption of any particular ‘Neolithic package’ in a given regional sphere demonstrates a specific socio-cultural process, but one which is unlikely to represent a general phenomenon in neolithization. As recently analysed—for example, in the NEOMAP project—there are several pathways to neolithization, including alternative histories of its constituent elements. Additionally, it has been pointed out that only analyses of differing Neolithic trajectories in different parts of the world will provide the complexity required for an understanding of cultural transformation in a global context (Uchiyama et al. 2014). This requirement not only enforces a ‘world prehistory perspective’, but can also lead to a closer, more reflective look at the traditionally modelled pathways from the Mesopotamian–Levantine core to the European continent (Zvelebil 2001). In the case of the eastern Mediterranean, including the Aegean sea, such a wider geographical perspective can supply important new data.

An important issue in this wider context relates to the potential elements that we understand as Neolithic parameters, which are usually not developed and adopted simultaneously, as assumed, for example, in the now outdated ‘wave of advance’ model (Ammermann and Cavalli Sforza 1984). One convincing explanation for the existence of such parameters within a regional but maritime perspective has been developed for the western Mediterranean world and is known as the ‘maritime pioneer colonization model’ (Zilhão 2001). This model describes the neolithization of the Iberian Peninsula as triggered by small groups of farmers from agricultural enclaves who sailed along the Mediterranean coast to the west. Although an attractive model, the question of where the acculturation of these seafaring populations triggering the neolithization of the Iberian Peninsula actually took place is nevertheless still highly debated (Chandler et al. 2005, pp. 781–786). ‘Leapfrogging seafaring colonists’, moving around the Mediterranean coast in groups, have been discussed as probable agents (Zilhão 2001). The regional context of cultural development sheds further light on the internal complexity of the neolithization process along the Mediterranean coastal zones. The regional and chronological diversity observed in the central Mediterranean early Neolithic may indeed be rooted in differing colonization and adoption processes, described as Adriatic and Cardial cultural zones, as discussed recently for Italy (Cruz Berrocal 2012). But a crucial point in this discussion has been the questioning of the interaction between local hunter-gatherers and newcomers in northern Italy, the two groups show no overlap in their regional distribution and display a lack of cultural indicators for contacts (Pearce 2013).

What is widely agreed is that the neolithization of the Mediterranean coastal zones is greatly affected by moving groups and seafaring connectivity. This is attested in various regions, but with diverse outcomes and differing local patterns of transformation (Zilhao 2001; Perlès 2001; Broodbank 2006). It is hence interesting to compare these Neolithic trajectories of the Mediterranean with other maritime neolithization models, for example the ‘far-eastern seaboard intensification phenomenon’ encompassing the coastal zones of China, Russia, Japan and Korea along the East China Sea and the Sea of Japan (Uchiyama et al. 2014). For these particular regions, it has been argued that the neolithization process is based on a complex pathway of transformation, detectable in societies with distinct and unique characters. Instead of fully developed and homogeneous Neolithic packages, what we observe is a variety of economic and material cultures, and these are based not only on the wide diversity of natural landscapes. Cross-cultural relations and long-distance exchange networks appear to play a crucial part in the rise of sedentary groups along the coastal zones within a long-term transformation of local societies (Uchiyama et al. 2014). The so-called aquatic, arboreal and coral reef Neolithic cultures in the Far East show a complex, and indeed highly diverse, neolithization in comparison to that so far established and accepted for the Mediterranean. In our view, however, a comparable diversity of economic and cultural patterns among seaboard cultures can also be observed in the Mediterranean. It is on this that we focus in the present contribution on the eastern Mediterranean coastal zone, a region which has strong cultural affinities to the Fertile Crescent.

The impressive recent advances in our knowledge and understanding of sites such as Göbekli Tepe in this core zone of neolithization demonstrate the impact on our scientific field of a constant stream of new primary archaeological data (e.g. Schmidt 2006, 2010; Özdoğan and Özdoğan 1989; Özbaşaran 2012). Although the scientific sensations associated with the monumental PPN sites are absent in regions distant from these Neolithic core zones, the picture in the periphery can also change dramatically with the advent of new sites and data. In the past decade, such fundamental and unexpected change in knowledge has been well illustrated in western Anatolia, where several Neolithic sites have come to light in different regions that were formerly *terra incognita* (Özdoğan, Başgelen and Kuniholm 2012b; E. Özdoğan 2015).

The new data not only sheds new light on the Neolithic in general, but also offers for the first time the possibility of integrating the vast areas of western Anatolia into the discussion of Neolithic trajectories (Hauptmann and Özdoğan 2007; Özdoğan 2010, 2011a, 2014; Perlès et al. 2011; Weninger et al. 2014; Clare and Weninger 2014; Çilingiroğlu and Çakırlar 2013). Due to its topographical location between Central Anatolia, the Aegean and southeast Europe, the region has long been of particular interest for modelling the diffusion of the Neolithic from the core zone to the west and northwest. Note that, by avoiding the use of modern state and artificial border terminology (Bar Yosef 2014), we are following the established definition of the Neolithic core zone, which is also known as the *Golden Triangle* and by the cultural–geographical terms *Upper Mesopotamia* (including southeast Anatolia), *Levant* and *Central Anatolia* (Hauptmann and Özdoğan 2007; Özdoğan, Başgelen and Kuniholm 2012a, p. 279).

However, detailed studies of recently-excavated sites have made clear that the majority of Neolithic sites in western Anatolia can be defined culturally as Late Neolithic and date no earlier than 6500 calBC, mostly later (Özdoğan, Başgelen and Kuniholm 2012b, 2013; Weninger et al. 2014; Brami 2014). Additionally, it has become clear that the different western Anatolian sub-regions of Marmara, Lake District and the Aegean coast mainly show an already developed Neolithic way of life (Krauß 2011). Without discussing all models in detail, recent publications generally include western Anatolia as peripheral to the core zones in terms of chronology as well as cultural development (Özdoğan 2014; Weninger et al. 2014; Arbuckle et al. 2014). However, the relationship of Neolithic transformation in western Anatolia to the influence of the core zone is not in dispute. When it comes to potential trajectories, the distribution of the Neolithic way of life in western Anatolia is most often described as migration from inner Anatolia via terrestrial routes to the north and west (e.g. Özdoğan 2007, 2010, p. 885; Lüning 2007; Çilingiroğlu and Çakırlar 2013), for which there are various possible modes, such as leapfrog movement, moving frontiers, transfer of knowledge, acculturation or infiltration (Guilaine 2007; Özdoğan 2010, p. 884; Thissen 2000; Reingruber 2011; Düring 2013). A maritime route for neolithization into the Aegean has also been discussed by several scholars (Perlès 2001; Broodbank 2006; Reingruber 2008, 2011; Kotsakis 2001), with the main focus on the development of the Greek mainland and the Cycladic islands.

The new excavations at Çukuriçi Höyük on the central Aegean coast of western Anatolia lead us to a re-evaluation of these models, and especially of those that put specific focus on the routes of neolithization into the Aegean basin. As will become clearer in the course of this paper, the newcomers show a strong maritime affinity, already visible in their established knowledge of Aegean obsidian sources, but in particular in their intensive procurement of marine food, and their overall unexpectedly high dependence on such food sources. Hence, in addition to established inland routes, we propose an additional maritime colonization of the western Anatolian coastal zone around 6700 calBC, via routes from the eastern Mediterranean to the eastern Aegean. The knowledge of such long-established maritime networks in the Aegean basin as well as in the eastern Mediterranean must, we assume, have been an important component of the cognitive models used by the newcomers reaching the western Anatolian coast; indeed this knowledge would have been a central part of their world view. Comparison of new radiocarbon ages from Çukuriçi Höyük with the previously established site-chronology of Ulucak demonstrates that the newcomers are the first farming and herding societies in this region. Since both settlements are without forerunners, they are considered pioneer sites.

## Early Holocene Mobility and Maritime Networks

If we consider a wider region in the Aegean, including its islands and coastal areas, it is possible to discern intensive sea travel by early Holocene mobile groups (Galanidou and Perlès 2003; Broodbank 2006; Reingruber 2011; Dawson 2014; Gurova–Bonsall 2014). New Palaeolithic and Mesolithic sites like Ouriakos at Lemnos (Efstratiou et al. 2014) or Stelida at Naxos (Carter et al. 2014) have been detected in these Aegean regions and complement recent basic research by Reingruber (2011, p. 294, Fig. 4) and Dawson (2014). Some of the sites show evidence of early Mesolithic fishing in the Aegean, reflected in exploitation of a rich ichthyofauna such as that found in Franchthi Cave and Cyclops Cave (Youra) (Rose 1995; Powell 2003; Stiner and Munro 2011, p. 627).

Early and ambitious maritime activity particularly turned into systematic seafaring at the Late Pleistocene—Early Holocene transition, as convincingly argued by Broodbank (2006, 2013). We follow his idea of the constitution of, and early-Holocene starting point for, trans-Mediterranean societies and the origin of seaborne networks (Broodbank 2006, p. 208). The pre-Neolithic sites not only reflect seafaring to various Aegean islands (the Cyclades, Crete, Dodecanese/east Aegean, Sporades), but also a systematic procurement of Melian obsidian, at least within a regional network (Reingruber 2011, p. 301, Fig. 14; cf. the intensity of networks in later periods analysed by Knappett et al. 2008). This regional Mesolithic network reaches at least as far as Kerame (Ikaria) in the eastern Aegean, facing the centre of the Anatolian Aegean coast. It has already been pointed out that early Holocene groups used the same obsidian source on Melos [Milos] as did early farming communities in the Neolithic period (Perlès 2001; Connolly 2008; Broodbank 2006; Reingruber 2011). Furthermore, this systematic obsidian procurement implies essential knowledge of sea routes, including currents, winds, natural harbour locations and freshwater sources, along with the particular location of the obsidian source on Melos itself. These expeditions are convincingly defined as ‘risky, dangerous journeys, requiring greater skill at manoeuvring craft in different conditions and over a longer duration, predictive and navigational knowledge of currents and winds, and more extensive mental maps of land- and seamarks’ (Broodbank 2006, p. 210). Finally, in our view, it is more than likely that mobile maritime scouting would have led to the discovery of the best obsidian source in the Aegean (Cherry 1981). Any initially even minor importance of seafaring knowledge would (or at least *could*) have been rapidly amplified via positive socio-cultural feedback.

On the other hand, this ‘package of seafaring knowledge’ could hardly have been introduced into the Aegean via contacts of the early Neolithic farming communities. At the centre of the Anatolian Aegean coast, such contacts start no earlier than 6700 calBC; perhaps around the same time in Crete (Reingruber 2008; Broodbank 2006); and c. 6500 calBC on the Greek mainland (Reingruber 2008; Reingruber and Thissen 2005; Perlès 2001). On the Cycladic and Dodecanese islands, the contacts start as much as 1500 years later (Broodbank 2000; Sampson et al. 2002). The absence of sites between the Mesolithic and the Early Neolithic inhibits the development of models with direct transfer of local maritime knowledge to the incoming western Anatolian farmers and herders.

As already mentioned, the early Aegean networks were not an isolated phenomenon, but show links with other networks in the eastern Mediterranean (Broodbank 2006). Cyprus had been reached by mobile groups from the Epipalaeolithic onwards (Peltenburg 2004). The PPNA and PPNB animal remains from the island indicate, albeit again indirectly, fundamental nautical knowledge and not least the construction of boats to reach Cyprus from the mainland (Knapp 2013). Open sea crossing was possible over distances of c. 70 km between Cyprus and the Anatolian coast, or c. 100 km from the Levant (Broodbank 2006, p. 209). The Neolithic settlements dating from the 10th to the 8th millennium yielded a variety of archaeozoological finds, but definitely including fish bones, with the fish representing littoral species like sea bream (*Sparidae*) and groupers (*Epinephelidae*) as well as pelagic species like tuna (*Thunnus* sp.) and mackerel (*Scomber* sp.) (Desse and Desse-Berset 1999, 2011; Bar-Yosef Mayer 2014).

The mammalian remains from Neolithic Cyprus indicate the occurrence of animals not native to the island, and this finding allows us to enlarge the discussion of the seafaring capabilities of PPN people. The earliest Neolithic stages are dominated by suids, interpreted as introduced wild boar which was hunted and managed (Wasse 2007; Vigne et al. 2003). The early forms of goat and sheep reveal an unstable status, with the domestic ungulates still showing morphologies of the wild ancestors (Vigne et al. 2011). The size of cattle indicates the introduction of already-domesticated animals, while fallow deer, also introduced, were never domesticated by the PPN settlers on Cyprus (Vigne et al. 2011).

Aside from the import of domesticated animals and subsistence strategies, the archaeological material culture is linked to the core zone in the Levant and upper Mesopotamia, reflecting early seafaring (Broodbank 2006, p. 209; Peltenburg 2004). Besides the state of nautical knowledge, the pattern of ichthyofaunal composition suggests the development of a PPN regional network in the eastern Mediterranean that, from our perspective, can be compared with the Mesolithic Aegean.

The Anatolian networks correspond to the distribution of obsidian blades produced in specialized workshops, for example Kaletepe-Kömürcü, situated at Göllü Dağ, one of the best-known Cappadocian obsidian sources. This obsidian network seems to have been established during the PPNB within the wider region of Anatolia, the Levant and Cyprus. Earlier published data show that in the 9th millennium calBC obsidian from Cappadocia was distributed as finished products, that is, standardized obsidian blades, to sites such as Dja’de, Mureybet, Tell Halula, and finally Shillourokambos in southern Cyprus (Binder and Balkan-Atlı 2001, p. 12). In the early stage of research, two possible routes for obsidian procurement from Cappadocia have been suggested: transfer via the Levant; and the maritime route directly along the Anatolian coast (Guilaine et al. 1997, p. 111). Recent obsidian distribution studies at certain Cypriot sites focus on the 9th millennium calBC. They shed light on a potential regional network within the island, in contrast with previous publications indicating direct import from the obsidian source in Cappadocia. Discoveries of large amounts of obsidian with Cappadocian provenance at Akanthou-Arkosyko/Tatlısu-Çiftlikdüzü meant that Shillourokambos was no longer the sole site with obsidian in the Cypriot Pre-Pottery Neolithic (Şevketoğlu 2006). The location of Akanthou on the northern coast of Cyprus, and the significantly higher amounts of obsidian in comparison to other contemporaneous sites on the island, have been used to argue for Akanthou as a possible gateway community for obsidian import from Anatolia and its further distribution within Cyprus (Şevketoğlu and Hanson 2015; Clarke 2013). The special geographical location of Akanthou on the northern coast of Cyprus implies the shortest distance to the obsidian source in Cappadocia. The transport route was along the southern coast of Anatolia, and attests to Mediterranean seafaring over short distances for the purpose of raw material procurement.

Recent scientific results on the Aegean and the eastern Mediterranean maritime networks reveal early Holocene societies with a package of seafaring skills and knowledge of distant obsidian sources. In accordance with Broodbank’s theory of the early Holocene origin of trans-Mediterranean societies (Broodbank 2006), territorial overlap and contacts with mobile groups exploring the sea of both regions seem plausible. It is most likely that it was within this potential contact zone—probably around the southeast Aegean and southwest Anatolian coast—that the transfer of the package of seafaring knowledge took place. However, the new arrivals at the centre of the Anatolian Aegean coast around 6700 calBC were apparently already equipped (along with their skills as farmers and herders) with this package of seafaring knowledge. Large amounts of obsidian from Melos at the pioneer site Çukuriçi Höyük (discussed in more detail below) are taken as a starting point for our concept of the newcomers’ maritime affinity. It is the procurement of large quantities of obsidian from this Aegean source, in combination with aquatic subsistence, that has triggered the present discussion of the pioneering character of the settlement at Çukuriçi Höyük, in its earliest levels.

## Maritime Versus Terrestrial Colonization

Discussion of the beginning of sedentism at the centre of the Aegean coast of Anatolia obviously first requires a close look at all the earliest sites in the region, paying particular attention to characterizing what we may call the ‘pioneer settlements’. As for the overall neolithization of the Aegean, although there are several sites which exhibit a fully developed Neolithic lifestyle in the region after 6500 calBC (cf. below), so far only Ulucak VI and Çukuriçi Höyük XIII–XII are sufficiently early, as attested by stratified sequences of ^14^C age estimates measured on short-lived samples, to qualify as potential ‘pioneer settlements’.

We still lack clear indicators for a period of transformation from Mesolithic, Epipalaeolithic or even PPN groups to Early Neolithic societies at the centre of the Anatolian Aegean coast. The absence of earlier populations in this particular region has been attributed to the fundamental change in the landscape since the early Holocene, which complicates the identification of sites (Lichter 2007). Indeed, the two pioneer sites in our focus, Ulucak and Çukuriçi Höyük, are known only due to their later accumulation as tell sites, with cultural deposits rising above the massive alluvium. However, the pattern of land choice by pioneer farmers in the central and western Mediterranean might be comparable to the process in other parts of the central Aegean coastal zone of western Anatolia (cf. Pearce 2013). In this context, the lack of Mesolithic groups (that is, precisely the absence of other populations) might have been an important reason for the newcomers’ choice of this area. This pioneer-based choice of an empty landscape might indeed explain the absence of any traces of interaction between hunter-gatherers and early farmers, as has more than once been pointed out (most recently by Çilingiroğlu and Çakırlar 2013). Moreover, the wide landscapes of western Anatolia were not empty as a rule, as evidenced by the occurrence of seasonal hunter-gatherer sites in northwest and southwest Anatolia (e.g. Ağaclı and Gümüşdere in the northwest, or Öküzini and Karain in the southwest). So far, there are no visible adaptive processes that might help in understanding the earliest implementation of farming and herding in the middle of the Aegean Anatolian coast in the Epipalaeolithic or PPN. Only assuming—without data—that new subsistence strategies and technologies had indeed been adopted by local populations through interaction with other groups, we might expect the occurrence of similar patterns of transformation and adaptation in the founding horizons of both Ulucak and Çukuriçi. However, it is the lack of any discernible process of Neolithic transformation that best supports our model: that of the conscious choice of an empty landscape by the newcomers. Beyond plausibility considerations, what this hypothesis requires is more robust data from modern field investigations, including environmental surveys purposely undertaken to rule out the presence of former populations in these particular landscapes.

So far, what is known is that the two earliest settlements (Ulucak Phase VI and Çukuriçi Phase XIII) both represent fully developed Neolithic farming and herding communities, and that both sites were most probably colonized by newcomers from outside the area. In addition to the traditional archaeological model of inland migrations from east to west, and without ruling out the existence of parallel pathways for such movement, we will now argue for an additional maritime route, at least for one of the two pioneer sites on the Aegean coast of western Anatolia. We now turn to a discussion of the archaeological evidence and present the oldest settlement of Çukuriçi Höyük in detail, including new and unpublished aspects like chronology, materiality, subsistence and technology.

### Two Pioneer Sites: Ulucak VI and Çukuriçi XIII

#### Overview of the Pioneer Phase at Ulucak VI

Ulucak VI represents a long-term and extensively-excavated founding settlement of a tell with remarkable features already published and discussed by Çilingiroğlu and Çakırlar (Çilingiroğlu 2011; Çilingiroğlu et al. 2012; Çilingiroğlu and Çakırlar 2013). Aside from pits and circular hearth installations, the remains of rectangular architecture—two separate buildings made of compacted mud-walls with red plaster on the floors and surviving inner walls—have been recovered. According to Çilingiroğlu and colleagues (Çilingiroğlu et al. 2012, p. 153; Çilingiroğlu and Çakırlar 2013, p. 23), the radiocarbon dates on short-lived samples from Ulucak VI provide calibrated ages in the range 7040–6470 calBC (95%-confidence). The excavators describe the lowermost Neolithic deposits at Ulucak as reminiscent of PPN features in central parts of Anatolia, a phenomenon also detectable at Çukuriçi Höyük (see below).

However, within the wide and statistically valid range, any potential socio-cultural relationship to PPN cultures further east cannot be confirmed by a detailed site-to-site comparison between Ulucak, Çukuriçi and other Turkish Neolithic sites based on available ^14^C dates (Clare and Weninger 2014). A chronological gap between the PPN further east and the pioneer sites Ulucak and Çukuriçi on the central Aegean coast of western Anatolia is obvious, and might be related to the later colonization at least of the latter. Neither can we confirm the restriction of the foundation date for Ulucak to an age value of ~7000/7040 calBC (Çakırlar 2012; Arbuckle et al. 2014), which is biased by simply taking the older side of the calibration plateau ~7000–6700 calBC as an indication of the oldest expected foundation date. Just as for Çukuriçi XIII, the ^14^C evidence would instead suggest an incipient occupation of Ulucak VI no earlier than the late Early Pottery Neolithic period. When based on stratigraphic wiggle-matching, with error analysis based on Monte Carlo procedures (Benz et al. 2012), the available N = 38 ^14^C dates for Ulucak levels VI–IV provide a chronology with a foundation date of 6630 ± 32 calBC (95%-confidence) (Weninger et al. 2014, p. 19, Fig. 12). As a result, we infer that Ulucak was founded approximately contemporaneously with Çukuriçi Höyük, within a time span of some few decades.

The deep and large deposits of Ulucak VI show a quite limited assemblage of material culture, with chipped stones (including a very small amount of obsidian) and bone tools with elements comparable to the Çatalhöyük bone tool assemblages, as far as can be judged from the recent publication, which does not include detailed material studies (Çilingiroğlu and Çakırlar 2013, p. 23). It may be unsurprising that the inland settlement of Ulucak VI shows subsistence based on (well-established) farming and herding, whereas fishing, even for freshwater resources, played no role at all. Hunting of wild animals is attested, but described as playing a secondary role in the general community’s subsistence. The excavators argue convincingly for the leap-frog colonization model for this already-established agricultural society with technological skills (red plaster), in a context of groups seeking suitable new habitats (Çilingiroğlu 2011, p. 69; Çilingiroğlu and Çakırlar 2013). They suggest a terrestrial colonization by a movement of these groups along the east–west oriented Gediz Basin from the western Anatolian interior.

#### New Data for a Pioneer Phase at Çukuriçi Höyük XIII

**Fig. 1:**
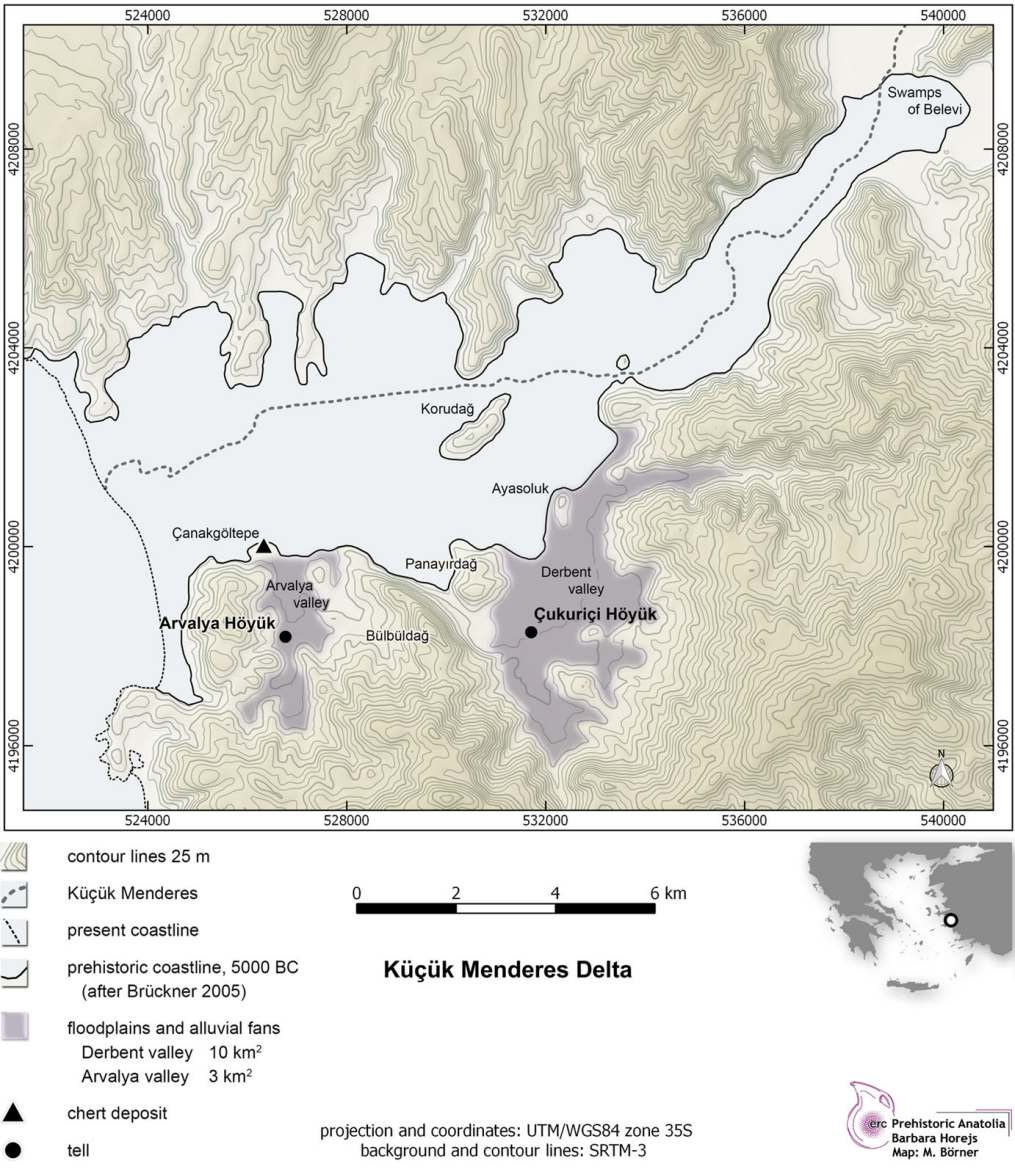
Çukuriçi Höyük (pioneer site) and Arvalya Höyük (Late Neolithic), situated close to the prehistoric lagoon (map: ERC Prehistoric Anatolia/M. Börner after Stock et al. 2013)

Çukuriçi Höyük is situated in a fertile basin of around 10 km^2^, on the southern shore of what in prehistory was a shallow coastal inlet characterized by lagoons and swamps, reaching nearly 20 km inland along the axis of what is now the Küçükmenderes river and its deltaic plain (Fig. 1). Such a micro-region was probably an optimal habitat for mobile groups seeking new land for settling, farming and herding. The encircling mountains, originally covered with deciduous oaks, provided not only an enclosed and therefore safe environment but also continuous fresh water draining into the plain in small creeks running east–west (Stock et al. 2013; Kayan 2014; Stock et al. 2015). The forested environment supplied essential natural resources (wood, wild game etc.). The array of rock types available on the Çukuriçi plain (e.g. marble, quartz, serpentine, chert) might have been a further asset to the newcomers (Wolf et al. 2012). The newly-founded site’s proximity to the prehistoric coastline (1.5 km) allowed direct access to the Aegean sea. The Çukuriçi settlers took advantage of this specific topography from the inception of the tell until its abandonment in the Early Bronze Age. (For an overview of all settlement phases in more detail, see, for example, Horejs et al. 2011).

**Fig. 2:**
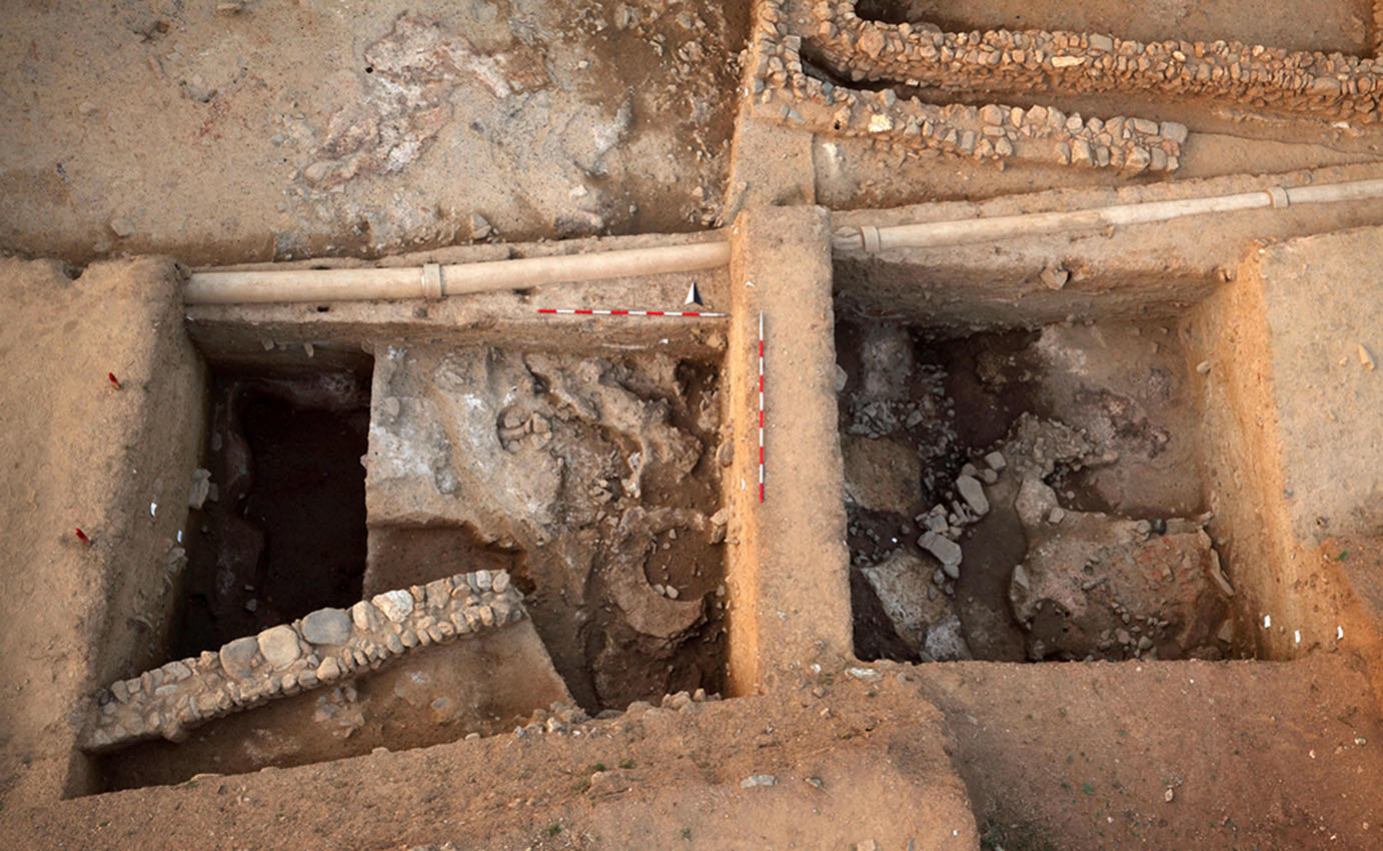
Deep sondages east and west in trench N6 with settlement Phases XIII–XI (photo: ERC Prehistoric Anatolia/N. Gail)

**Fig. 3:**
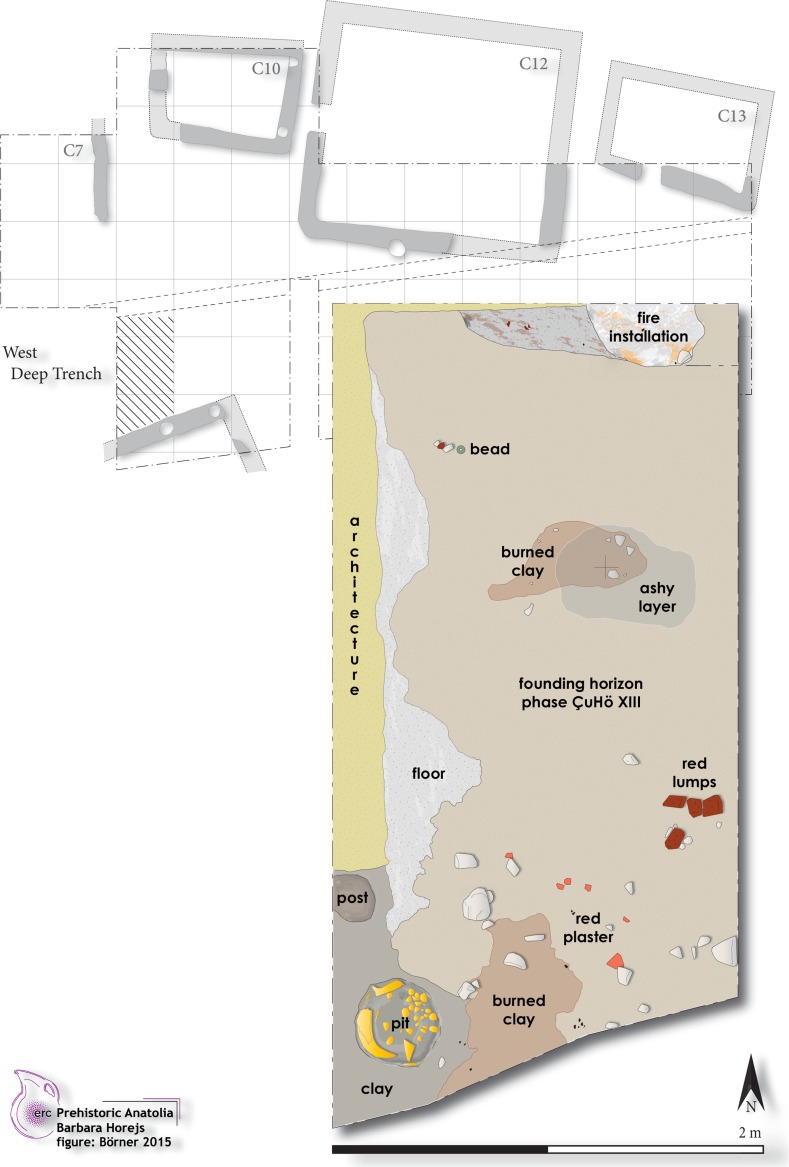
Archaeological remains (Complex 24) of founding phase XIII at Çukuriçi Höyük as a blown-up detail of the cross-hatched area with settlement Phase X and complete trench N6 in the background (plan: ERC Prehistoric Anatolia/M. Börner)

The first Neolithic settlement activities at Çukuriçi Höyük are attested through several drilled core probes, as well as by excavations within two deep trenches (Fig. 2). This limited area totalling 50 m^2^ provided three distinct phases, differentiated as ÇuHö XIII, XII and XI. Whereas Phases XII and XI could have been recovered in larger areas, ÇuHö XIII was unfortunately limited to a small trench of 2 × 4 m. Due to the good state of preservation and clear archaeological features, the recovered area nevertheless yielded secure information about the founding horizon ÇuHö XIII (Fig. 3). Domestic features of compacted pure yellowish clay walls with one associated post(hole) perhaps indicate an entrance situation, possibly extending into an enclosed inner area with a minimum size of 3 × 2 m or 6 m^2^. Within this building (Complex 24), a sequence of two or three lime-plastered floors were found, partially preserved in a deposit around 30 cm thick. The first and oldest floor constructed immediately above the natural soil is composed of clay with red plaster (layers of white lime painted bright red), preserved as small fragments only. Ashy layers and burnt clay mark a potential inside hearth, corresponding to the first use of this building, Complex 24. The deposit of several red lumps (presumably hematite) upon this first floor indicates storage of pigmenting substance within the building. The deposition of a horn core and other animal bones, molluscs, obsidian and charcoal inside a circular pit might indicate various activities of the Çukuriçi founding settlers. The deposition of different stone implements in the initial horizon, as well as in the following use levels within the building, including two other potential hearth installations on lime-plastered floors, distinguish Complex 24 as a domestic building.

**Fig. 4:**
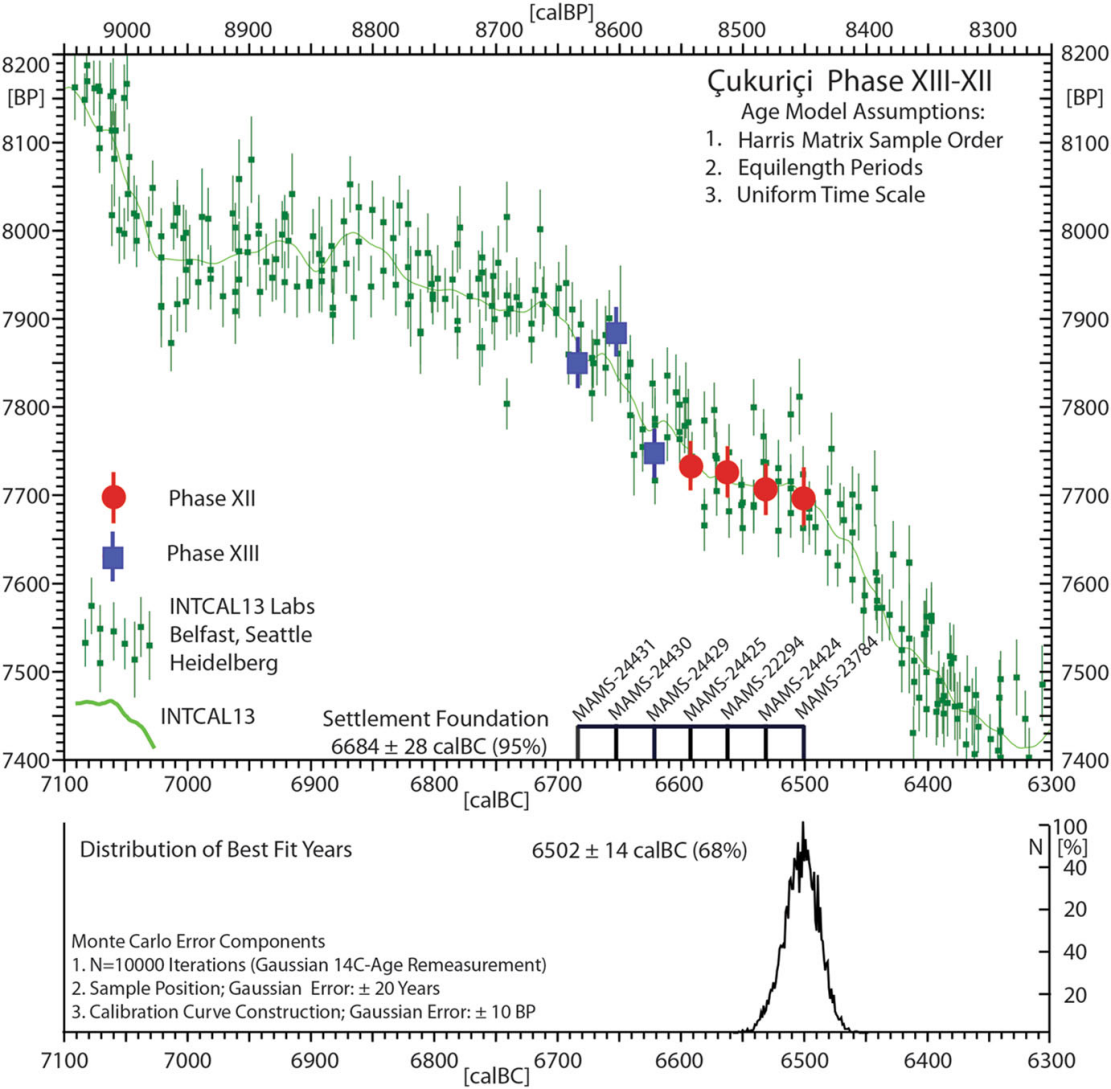
Radiocarbon dates (Table 1) on short-lived samples from Çukuriçi phases XIII–XII, analysed by Gaussian Monte Carlo Wiggle Matching (Benz et al. 2012). The dates are uniformly sequenced from old to young according to the stratigraphic position of the samples as recorded in the Çukuriçi Harris matrix

The absolute chronology of the initial Phase ÇuHö XIII is based on three radiocarbon dates, supported by another four dated samples from the subsequent Phase ÇuHö XII. They offer a robust sequence of seven datings in total, obtained from short-lived samples (Fig. 4; Table 1). Applying the method of Gaussian Monte Carlo Wiggle Matching (GMCWM) (Benz et al. 2012), the beginning of the settlement can be set at 6684 ± 28 calBC (95% probability). The sequence of the radiometric data additionally indicates a continuous use of the site in the two oldest settlement phases within a narrow window of around 180 years.

**Table 1:** AMS data of phases ÇuHö XIII and XII (ERC Prehistoric Anatolia/F. Ostmann and U. Thanheiser)

Lab-Code	^14^C-Age [BP]	±1 [BP]	δ^13^C [%PDB]	Calendar Age 68% [calBC]	Calendar Age 95% [calBC]	LOCUS	PHASE	SPECIES
MAMS-22294	7726	29	−36.6 (AMS)	6594–6508	6629–6480	Eil CuHö14/2296/11/1	XII	Grass
MAMS-23784	7697	33	−18.4	6587–6480	6597–6466	CuHö14/2208/11/1	XII	Cereal unidentifiable
MAMS-24424	7707	29	−20.5	6588–6500	6597–6472	EIL 1—2210/10/1 − 2	XII	Cultivated legume
MAMS-24425	7734	28	−22.7	6597–6508	6636–6485	EIL 2—2288/10/2 − 2	XII	Cultivated legume
MAMS-24429	7748	28	−20.9	6633–6515	6641–6503	EIL 6—2475/10/1 − 1 + 2	XIII	Wheat
MAMS-24430	7886	28	−18.4	6767–6657	6981–6645	EIL 7—2491/10/1 − 1	XIII	Cereal unidentifiable
MAMS-24431	7851	29	−16.7	6695–6642	6772–6608	EIL 8—2491/10/1 − 2	XIII	Wheat

GMCWM is an extension of the wiggle matching method developed by Weninger (1986), whereby the archaeologically sequenced ^14^C data are fitted to the calibration curve based on a Chi squared (χ^2^) test. By systematically expanding the sample sequence, along with stepwise re-calculation and comparison of the associated best-fit probabilities with the aim of identifying the optimal length and beginning/end of the sequence, this approach quite often immediately supplies a definitive answer, in the form of a unique value. What complicates matters, however, is the also typical existence of a multitude of alternative (either/or) best-fit solutions, the existence of which can be recognized by their equally high dating probability. Such alternatives may even exist, differently, for different parameters, such as sequence externally (begin/end ages), and sequence internally (phase-boundaries), as well as for requested combinations of such parameters (phase-spans). The existence of such ‘lock-in’ or ‘quantization’ effects is probably better known for single ^14^C ages. However, in our experience, they can be observed—and can have similarly large magnitude (~100 years)—for extended ^14^C-age sequences (Krauß et al. 2014). A full understanding of such effects has not yet been reached (cf. Weninger 2011, 2015). In the meantime, to support their identification, in the CalPal software version applied here, we now include a further optimization of previous Monte Carlo procedures. The previous procedure was to separately evaluate dating probability and dating precision, and to optimize both parameters simultaneously. The new procedures make further allowance for potential errors in the archaeological age-modelling, which is based on the (optional) introduction of a phase-internal (as well as the phase-wise) randomization of the sample order. Such random shuffling has its main use (next to measuring position and shape of the ^14^C-quantum states for extended sequences) in quantifying the dating errors achieved for individual sample positions (e.g. beginning/end and other boundaries). What remains unchanged is the basic approach, in which the initial sample order is provided on a metric scale (e.g. 0–100 U), which is then age-scaled based on an age-model (in the present case: equal length of the two Phases Çukurici XIII and XII). What is new is the refined error analysis, which is based on the above-mentioned phase-internal random shuffling of the sample order. Note that the specific sample order shown as ‘best result’ in Fig. 4 is actually representative for only one of many thousands of different sample orders that have been tested in order to measure the overall ‘age-model variability’. We have abbreviated the otherwise overly complicated age-model variability by providing the results with identical calibrated ‘+ values’ for beginning/end of the sequence, as given in Fig. 4.

### Material Culture at Çukuriçi XIII and its Relation to the Core Zone

**Fig. 5:**
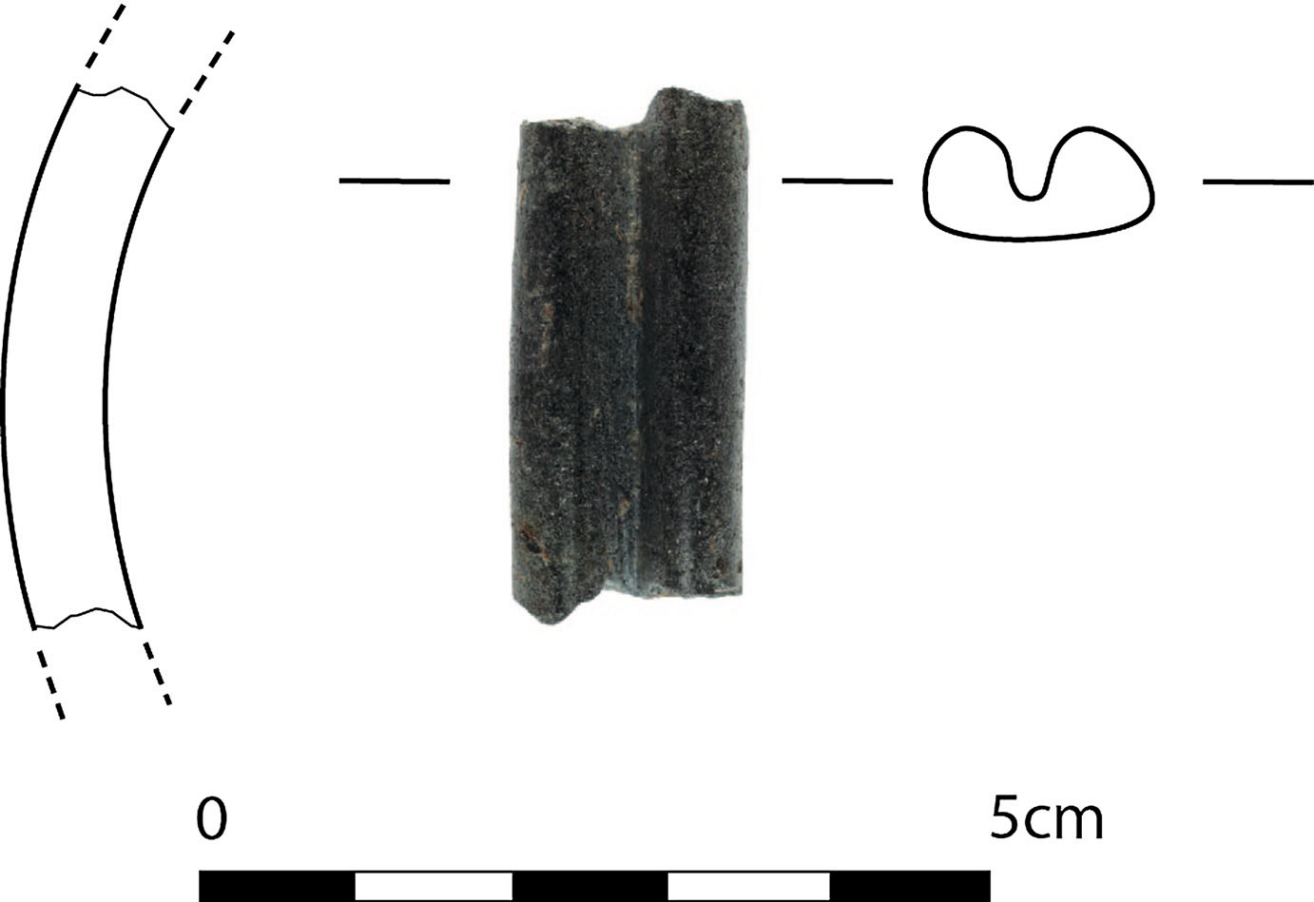
Polished stone bracelet with profiled and slightly convex section from Çukuriçi XIII (ERC Prehistoric Anatolia/M. Röcklinger)

The material assemblage of Phase XIII contains some stone implements and objects (such as a spheroidal ‘hand-stone’), including a polished bracelet made of potentially local mica slate (Fig. 5). The high-quality polished surface and the convex and V-shaped cross-section of the latter are reminiscent of the stone bracelets with convex and profiled sections usually known in late PPN contexts in the core zone, especially in the eastern extension of the Fertile Crescent, from Çayönü and Cafer Höyük in the north to Ali Kosh in the south (Kozlowski and Aurenche 2005, p. 197). A profiled bracelet made of obsidian from Aşıklı dating to the 8th millennium BC also seems to some extent comparable (Astruc et al. 2011). The pattern of distribution of the particular type of bracelet is obviously different from that of the southern Levantine ones with their triangular and sub-triangular sections (Kozlowski and Aurenche 2005, p. 196). Late PPN bracelet types from upper Mesopotamia offer the best comparison to the Çukuriçi fragment so far.

The set of beads from ÇuHö XIII is heterogeneous in terms of shape and materials. Malachite (lenticular with vertical piercing), black stone, mica slate, serpentine and sandstone (all disc-shaped) were used. Malachite beads are unknown in the Mesolithic Aegean, but common in the PPN core zone (e.g. Çayönü: Erim and Özdoğan 2011, p. 268, Fig. 74), as well as in Central Anatolia, for example in Aşıklı (Esin 1994). Aside from the particular material, the flat oval shape is observed frequently throughout the Fertile Crescent (e.g. Mezraa Teleilat: Özdoğan 2011b) and sometimes also in Central Anatolia, mostly in the 8th and 7th millennia BC (Kozlowski and Aurenche 2005, p. 185).

**Fig. 6:**
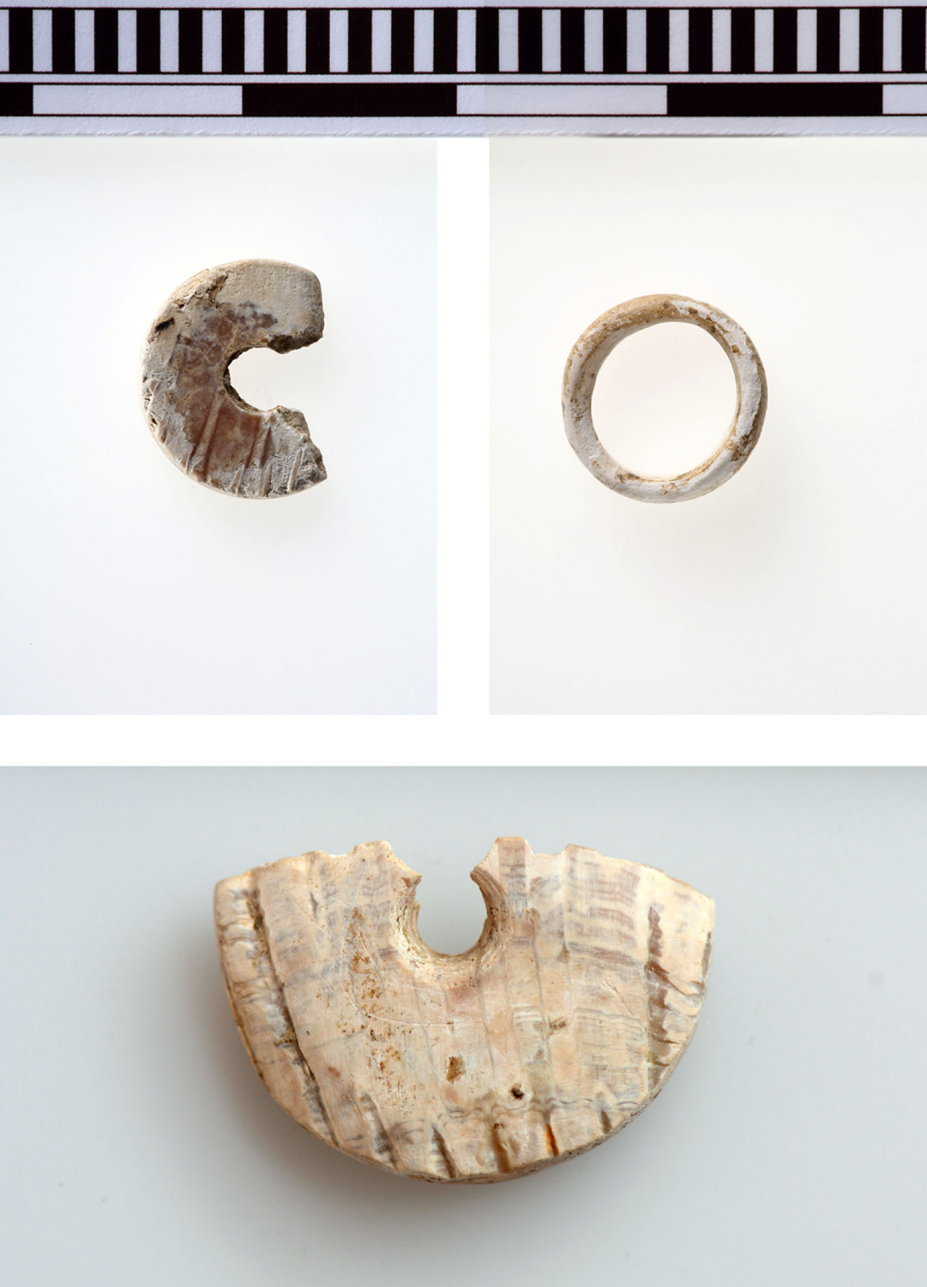
Two beads and a ring made of marine shells from Çukuriçi XIII (ERC Prehistoric Anatolia/N. Gail and F. Ostmann)

Two disc-shaped beads made of bivalve marine mollusc shell (Cardiidae: *Cerastoderma* sp., the common cockle), and a ring probably made of a spondylus shell, demonstrate the secondary use of marine shells for ornament production (Fig. 6). Such a practice disappears at Çukuriçi after 6500 BC. The masses of seashells, including spondylus, were simply deposited as consumption refuse throughout the settlement until the end of the Neolithic occupation around 5900 calBC, the end of Phase ÇuHö VIII. In total only nine ornaments (seven beads, one ring, one bracelet) are known from within the founding phase of the tell. Therefore, the evidence of three shell objects might indicate that the colonizers had strong maritime links, as reflected in their subsistence strategy (see below).

Bone tools in ÇuHö XIII include pins, awls, points, spatulas and smoothers, but the bone spoons common in the later Neolithic after 6500 BC appear to be absent. Four small and abraded sherds of varying fabric types, none larger than 3 cm maximum, appear to belong to this earliest, founder, layer. No evidence of later disturbance or markedly mixed deposits has been documented, and although processes of re-deposition within the tell mound could have displaced these, and although no diagnostic rim or base sherds were recorded in ÇuHö XIII, the use of pottery vessels by around 6700 BC would not be surprising. Thus it seems most likely that whilst ceramic vessels were present in the founder phase at Çukuriçi, they did not play a major role in the overall material culture.

#### Obsidian in Çukuriçi XIII and its Impact

The lithic material so far recovered from the earliest phase of the settlement occupation consists of 206 single pieces. Although the excavated area is quite limited and the sample size is relatively small, several features observed in the lithic assemblage of Phase XIII attest major differences between the foundation level and the following occupation phases in terms of chipped stone artefacts.

**Fig. 7:**
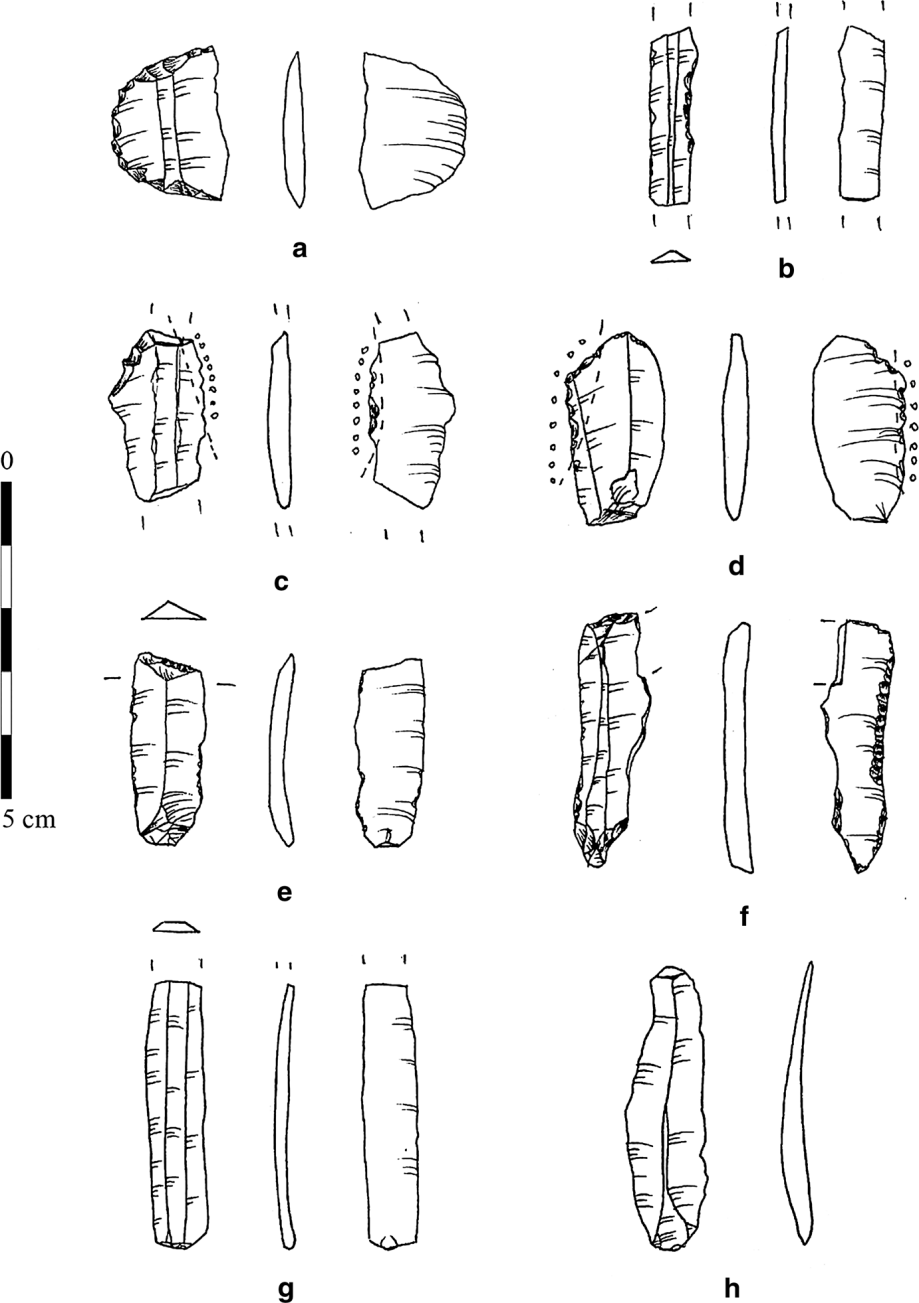
Lithics of CuHö XIII (ERC Prehistoric Anatolia/B. Milić)

The analysis of lithics at Çukuriçi Höyük showed that obsidian dominated raw material choices throughout the life of the settlement, reaching up to 86% among the assemblage of chipped stone artefacts in the Late Neolithic, and remained a stable feature for the duration of the occupation. A similar pattern with high amounts of obsidian is visible in the early Phases XI and XII, while the raw material ratio of the foundation Phase XIII presents a remarkable shift in raw material use (Table 2), with, for the first time, knapped quartz in varieties of smoky quartz, milky quartz and rock crystal occupying a significant place within the assemblage (Fig. 7). The low proportion of obsidian artefacts in Phase XIII (34%; see Table 2), in contrast with the prevalence of obsidian as a raw material, may not simply be a function of the small sample size, since the subsequent Phase XII shows the reverse situation with a similar number of artefacts and could thus indicate a systematic difference.

**Table 2:** Raw material ratios of knapped chip stones in early phases of Çukuriçi Höyük with highlighted amounts of obsidian (ERC Prehistoric Anatolia/B. Milić)

Phase	Phase XI	Phase XII	Phase XIII
Raw material ratio	**79**% (obsidian) 21% (chert)	**70**% (obsidian) 30% (chert)	**34**% (obsidian) 52% (chert) 14% (quartz)
Assemblage size	N = 2837	N = 305	N = 206

The initial results of Neutron Activation Analysis (NAA) conducted on a few sample assemblages indicate that obsidian from Çukuriçi Höyük originates from the Aegean island of Melos (e.g. Horejs and Milić 2013), with positive identification of both the Adamas and Dhemenegaki obsidian flows. It remains unclear whether the Melos obsidian outcrops predominate in the Çukuriçi obsidian-based technology. Additional provenance analysis involving pXRF, currently in progress, which will sample a rather larger number of obsidian artefacts at Çukuriçi Höyük may broaden our knowledge here. However, no major differences in obsidian techniques for blade production are visible, as the knapping follows the unidirectional system in a homogeneous way from the earliest phase of settlement occupation until the end of the 7th millennium BC.

#### Lithic Technologies in Çukuriçi XIII and the Question of the Early Pressure Technique

Preliminary results on the lithic technology from the earliest Phase XIII showed that tools were mostly obtained by percussion (Fig. 7h); however, blades with parallel ridges, slight curvature of profile, reduced thinness, pronounced short bulb and the absence of any impact point testify to the presence of pressure debitage among the assemblage (Fig. 7b, g).

The first appearance of the pressure technique in blade making in Anatolia is among PPNB assemblages of the mid to late 9th millennium in southeast and Central Anatolia, that is, at Çayönü Tepesi and the Kaletepe-Kömürcü workshop (Binder 2007; Balkan-Atlı and Binder 2012; Altınbilek-Algül et al. 2012). As has been suggested, the emergence of the Aşıklı-Musular-Çatalhöyük complex at the start of the 8th millennium BC effected the interruption of the remarkable specialized blade production based on pressure technique from the Kaletepe-Kömürcü workshop (Binder 2007). The firm establishment of a bidirectional technique in tandem with unidirectional knapping and unstandardized assemblages of blade-like flakes without pressure technique, marked the Aceramic Neolithic assemblages of Central Anatolia in the 9th and 8th millennia BC, for example at Aşıklı Höyük (Balcı 2013), and early stages of Çatalhöyük, with emphasized production of projectiles (Carter 2011).

A gap of almost two millennia in the use of pressure flaking in Central Anatolia, which began after the Kaletepe-Kömürcü workshop, was ended by the introduction of pressure blade making in the mid 7th millennium BC at Çatalhöyük (Conolly 1999). At Çatalhöyük, it is thought to be an adoption from southeastern Anatolia and/or the northern Levant, via re-established contacts with those regions (Carter and Milić 2013, p. 502). The first practice of pressure blade making at Çukuriçi Höyük and Çatalhöyük occurred at a similar time, although perhaps 200 years earlier at the former site. However, the distinction between the two sites consists in the major tendency for blade making at Çukuriçi Höyük from the beginning of the settlement, and the virtual absence of bidirectional techniques, while the first chipped stone assemblages of Çatalhöyük obviously have their roots in a different tradition. Çatalhöyük lacks homogeneity in its lithic industry, with various techniques used throughout the long occupation of the site (Carter 2011, p. 11). Besides the absence of Central Anatolian obsidian from Cappadocia at Çukuriçi Höyük during the Neolithic, and its common presence in later lithic assemblages of other 7th millennium BC sites in western Anatolia (Milić 2014, p. 288, Fig. 2), the striking difference in the lithic technology of the Aceramic and Pottery Neolithic of Central Anatolia, with the long tradition of bidirectional systems of knapping, clearly suggests that Çukuriçi Höyük belonged to a different sphere.

Basic modes of pressure-flaked blade manufacture—detachment by hand or shoulder pressure (according to Pelegrin 2012)—dating back to the founding Phase XIII of Çukuriçi Höyük, have been attested in both chert and obsidian, while several wider obsidian blades could argue for more complex modes of pressure debitage by the first settlers of Çukuriçi Höyük. In this case, more material from the founding phase is needed to determine the exact state of pressure flaking knowledge among incoming people, since a certain level of specialization in pressure flaking is observed immediately in the following Phase XII. However, to our knowledge, no pressure debitage has been detected in the initial phases of occupation of neighbouring Neolithic settlements, and therefore the earliest documented practice of the pressure technique on the western Anatolian coast appears to be around 6700 calBC at Çukuriçi Höyük. According to the investigation of detailed technological features of assemblages, two possible centres of origin for the pressure technique can be suggested here: first, southeast Anatolia; and second, eastern Mesopotamia, in particular northern Iraq, both belonging to the area known as Upper Mesopotamia (Milić and Horejs, in preparation). It remains unclear whether the onset of pressure blade making had to do only with groups who already possessed knowledge, or whether obsidian procurement was an additional trigger in its use from the beginning of the settlement. As mentioned above, Ulucak VI yielded only a very small amount of obsidian (Çilingiroğlu and Çakırlar 2013), and further technological studies of the material, investigating the presence or absence of pressure technique in the earliest phase of Ulucak, could help us understand whether the technology was dependent on the raw material, that is, on obsidian. Nevertheless, the presence of minor obsidian core preparation elements, together with some flakes and debris in Phase XIII, suggests knapping on the spot, rather than the import of prefabricated blades and tools from the source. However, it seems reasonable to suppose that the incoming settlers would have come into direct contact with the Melos sources through the agency of groups already circulating along the eastern Aegean coast. This would have facilitated the development of sufficient technical knowledge for at-source preparation of obsidian material by the settlers themselves. It seems, therefore, that the first people of Çukuriçi Höyük were integrated into the Aegean sea networks and probably first made contact with the island of Melos at the time of their initial movement into the region.

The earliest phase of the settlement again showed slightly different typological characteristics in tool use and manufacture. Assemblages from later phases showed a low volume of retouched tools. Pressure debitage yielded masses of blades, which completely dominate the Neolithic assemblages, and in most cases do not show retouch or macroscopically visible traces of use. In contrast, Phase XIII showed 25% retouched or used tools among the chipped stone artefacts. Among the repertoire of tools here, retouched bladelets and micro-blades are most common, followed by end-scrapers on blades and flakes, and several sickle implements (Fig. 7c, d). For the first time at Çukuriçi, a multidirectional core for micro-blades and flakes in smoky quartz was documented in the earliest phase. The rare but significant combination tools where retouch clearly indicates multifunctional use (Fig. 7f) were curated for long periods, as shown by indices of repair and post-breakage reshaping. This kind of curation and reduction could indicate a scarcity of raw material in the founder phase, and contrasts with the overall characterization of later phases with a limited and distinct set of types at a time when raw material procurement routes were well established.

**Fig. 8:**
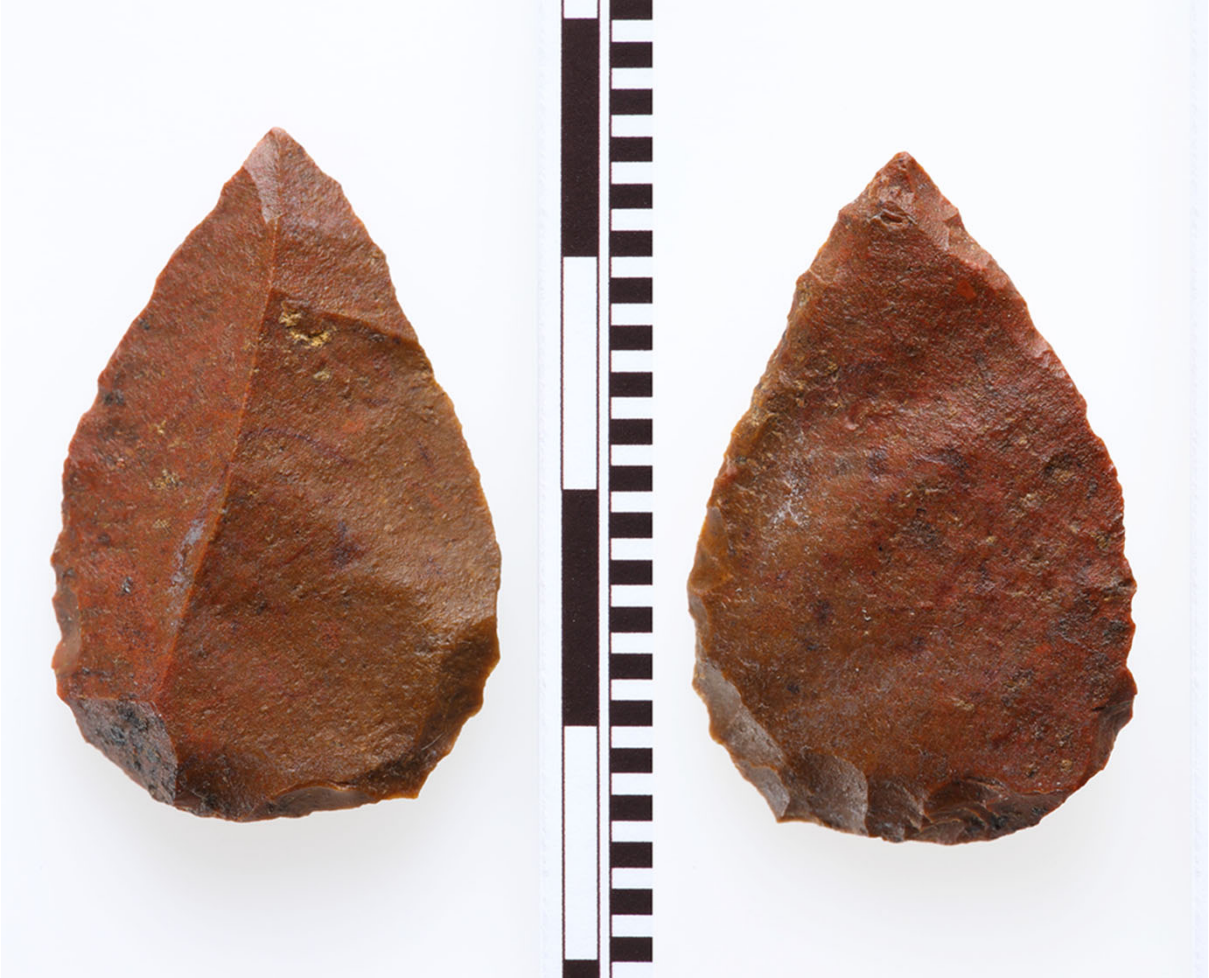
Jasper ‘foliate point’ from Çukuriçi XIII (ERC Prehistoric Anatolia/N. Gail)

Finally, unique finds among the chipped stone artefacts that marked Phase XIII seem to invite supra-regional comparison. So far, no points or arrowheads have been found in the Early Neolithic of the Aegean region, either on the Greek mainland (Perlès 2005), or the western coast of Turkey. It has been broadly accepted that during the final stage of migration of the people from the ‘eastern’ areas regular arrow points were replaced by sling missiles (Özdoğan 2002). However, it seems that the sling missile first appears in the period after 6500 calBC at the centre of the western Anatolian coast, within the Late Neolithic of the region (Horejs, 2016). The assemblage of the founding phase of Çukuriçi Höyük has yielded a small point, made in red jasper, with retouch on the tip of the ventral side, and characteristic inverse retouch on the bottom of both dorsal and ventral sides for inserting it into the shaft, with remains of as yet unidentified residue (Fig. 8). Examination of all Neolithic contexts where points have so far been found, across the entire area of the Aegean, Central Anatolia, Upper Mesopotamia and the Levant (e.g. Kozlowski and Aurenche 2005), reveals similarities exclusively with PPN settlements in southeastern Anatolia, that is, the PPNA of the Upper Tigris basin. Small points characterized by a round retouched base, found at PPNA sites at Hasankeyf and Demirköy, falling into the category of microlith foliates (Miyake et al. 2012, p. 6; Rosenberg and Peasnall 1998, p. 205; Yakar 2000, p. 483), are typologically comparable with the Çukuriçi point from the founding phase. In terms of size and shape, the small point in red jasper from the founding phase could therefore, in our opinion, be designated a foliate point, and so understood as a descendent of earlier eastern types.

Assessing the currently available evidence it seems that the distance in time between the regions we refer to in this section could be explained in terms of transmission of ideas through generations of people living in and finally moving out of the core zones. There are gaps in our current knowledge, however, which prevent us explaining why specific elements, very prominent in one time period, are lacking from the following period, and then show up much later in the same forms but different contexts.

Another lithic artefact documented for the first time in Phase XIII at Çukuriçi is a retouched rock crystal tool (Fig. 7a); this has a lunate or segment shape, characteristic of a microlithic industry. However, taking account of the sizes of the original microliths, this find could be characterized as a ‘broad segment’, of a type already proposed at several Neolithic settlements in the easternmost regions, such as Iran, with widths as great as 35 mm (Abe 2011, p. 165). It is known that similar forms of geometric microliths were in use as transverse arrowheads and barbs since Palaeolithic times (Lombard and Phillipson 2010), particularly among the Epipalaeolithic communities of the Levant, where use-wear analysis and breakage types attest to specific ways in which they were attached to shafts (e.g. Yaroshevich et al. 2011). The context of the Çukuriçi quartz find, located near another jasper point, could suggest the same use for both tools, bearing in mind their exclusive appearance in the foundation phase. The absence of a pre-Neolithic sequence in the region, on the one hand, and the lack of exact types in the Epipalaeolithic in the wider region on the other hand, for example at Girmeler (Takaoğlu et al. 2014), speaks in favour of an introduction of such elements by people migrating from another region, with possible adaptation due to the use of local raw materials.

### Subsistence Strategies in Çukuriçi XIII Embedded in a Diachronic View

Preliminary results of archaeobotanical studies indicate that both (unidentifiable) pulses and cereals are consistently present throughout the site from the Phase XIII founding horizon onwards. A strict sampling regime was adopted for the recovery of archaeobotanical remains, covering all features (for methodology see Horejs et al. 2011). However, plant remains are few and far between, usually only one item per ten litres of soil. The state of preservation is extremely poor and often does not allow identification beyond family or genus level. This stands in strong contrast to the richness of archaeozoological remains. Barley (*Hordeum vulgare*) is the dominant cereal crop, followed by emmer wheat (*Triticum dicoccum*). Grape (*Vitis vinifera*) is also occasionally present. Characteristic of all features is the absence of cereal-processing remains, namely chaff and weeds. Since the solid rachis fragments usually preserve well, their conspicuous absence may indicate that the harvest was processed in areas not yet excavated, or that the end product of cereal processing, the grain ready for consumption, was brought from an area outside the settlement. However, the initial botanical results generally indicate farming activities in the earliest Çukuriçi settlement Phases XIII–XI.

**Fig. 9:**
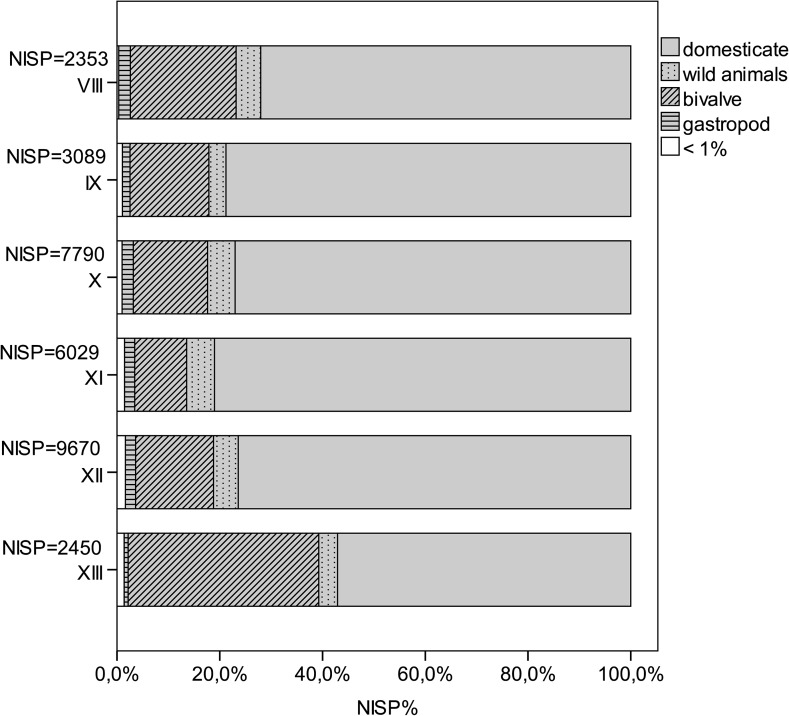
Quantification of the animal remains from Neolithic Çukuriçi Höyük (ERC Prehistoric Anatolia/A. Galik)

**Fig. 10:**
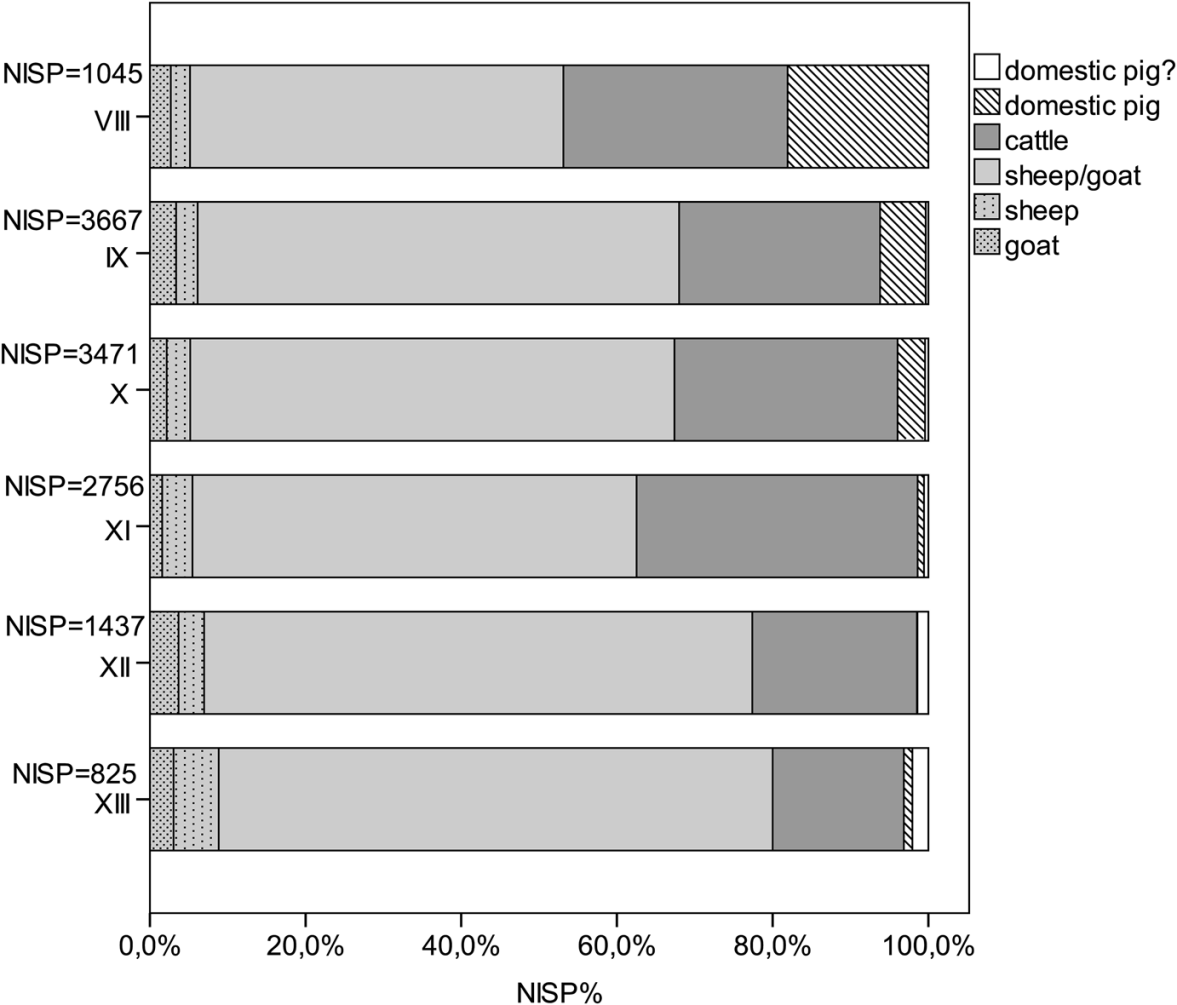
Quantification of domesticates from Neolithic Çukuriçi Höyük (ERC Prehistoric Anatolia/A. Galik)

Preliminary results for the earliest faunal assemblages from Phase XIII to XI vary in terms of their terrestrial and maritime archaeozoological remains (Fig. 9). Hand-collected material only is used in Fig. 9 to provide comparative and methodologically-unbiased data, because the investigation of sediment samples differs considerably between the phases. Therefore, Fig. 9 does not contain the little evidence of echinodermates and crustaceans from these phases. The amount of shell declines from Phase XIII (~38%) to slightly over 10% in Phase XI, and the proportion of terrestrial remains increases from about 60% in Phase XIII to about 85% in Phase XI. In the early phases, ovicaprines dominate domesticates with more than 80% in Phase XIII and 63% in Phase XI (Fig. 9). Cattle make up less than 20% in Phase XIII, rising to 40% in Phase XI. Pig appears only in very low numbers in all three phases, and as juvenile bones only, with very few remains having the shape and size of domesticated pigs (Fig. 10). However, in these earliest phases of settlement (XIII–XI), the number of large individuals resembling the wild ancestors of domesticates, such as ovicaprines, cattle and pig, is higher than in the succeeding phases. Fallow deer represent the main part of the wild animals, besides hare, wild boar, some red deer, aurochs and wild cat (Table 3). There are a few unidentifiable bird bones, which include a single instance of raven, in the earliest phases (Fig. 9). Some of the fish bones indicate littoral fish, such as sea bream, sea bass, groupers and bluefish. Others indicate pelagic fish such as tuna. Besides these, remains of chondrichthyes, including large stingray stings, are also known. Bivalves are predominantly lagoon cockles (*Cerastoderma glaucum*). Other burrowing bivalves are the corneous wedge clam (*Donacilla cornea*), venus shell (*Venus verrucosa*), carpet shell (*Ruditapes decussatus*) and the noble pen shell (*Pinna nobilis*). Hard-substrate-preferring bivalves comprise about 40% of the material within the earliest phases and are ark clams (*Arca noe*) and the bearded ark clam (*Barbatia barbata*); mussel (*Mytilus gallo*-*provincialis*); oyster (*Ostrea edulis*); spondylus (*Spondylus gaederopus*); and two species of boring bivalves: the date shell (*Lithophaga lithophaga*) and the piddock (*Pholas dactylus*). Only limpets (*Patella* sp.), purple snail (*Hexaplex trunculus*) and ceriths (*Cerithium vulgatum*) are of some importance amongst a great variety of archaeologically-documented marine gastropods. Most of the molluscs live in shallow lagoonal areas and can be easily collected, for instance, crustaceans and echinodermates. Spondylus, ark clams and oysters have to be actively removed from the sea bed by diving. The fish can be caught in the littoral zone of the Mediterranean next to the settlement. However, although fishing for tuna probably does not necessitate innovative fishing equipment it certainly requires a higher level of organization and experience in when and how to catch the fish.

**Table 3:** Quantification of the wild animal remains from Neolithic Çukuriçi Höyük (ERC Prehistoric Anatolia/A. Galik; (?) indicates that some of the Bos primigenius remains are at intermediate position between Aurochs and domesticated cattle)

Phases	*Erinaceus europaeus*	*Lepus europaeus*	*Vulpes vulpes*	*Ursus arctos*	*Marters* sp.	Mustelidae	*Meles meles*	*Felis silvestris*	*Sus scrofa*	*Cervus elaphus*
VIII		4	4					1	18	5
IX		13	3		1	2	1	2	17	7
X		56	11	2				1	36	6
XI	1	53	11					1	26	
XII		22	11		1			2	22	
XIII		14	4						9	4
Total	1	162	44	2	2	2	1	7	128	22

Phases	*Capreolus capreolus*	*Bos primigenius* (?)	*Dama dama*	*Dama* Antler	Cervidae	*Panthera pardus*	Carnivore	Delphinidae	Total
VIII	1	4	30	1	1	1			70
IX		3	109	1				1	160
X		1	142	2					257
XI		4	92	4			1		193
XII			103						161
XIII		1	37		4				74
Total	1	13	513	8	5	1	1	1	914

These archaeozoological patterns identify the first settlers at Çukuriçi Höyük as farmers and herders with an additional strategy of maritime-based nutrition. The large amount of marine shell and the numerous fish remains indicate a different subsistence pattern than in Ulucak VI, and demonstrate that the first settlers at Çukuriçi Höyük were experienced fishers from the outset. These settlers may have brought their skills with them, from areas where we know that traditional maritime subsistence was well-established in the Epipalaeolithic and PPN, perhaps Cyprus, the Levant or the Mesolithic Aegean (e.g. Franchthi). Skills for maritime exploitation could hardly have been transferred to the Aegean coast by inland Anatolian herders, especially as this subsistence pattern was already well-established in former coastal communities in other regions. However, the combined subsistence strategy of farming, herding, fishing and shell fishing in the Çukuriçi Höyük founding horizon is so far the oldest evidence in western Anatolia.

## The Fully Developed Neolithic at the Centre of the Anatolian Aegean Coast after 6500 BC

After 6500 calBC or slightly later, an increase in settlement is detectable at the centre of the Anatolian Aegean coast (Horejs 2016, Fig. 3), as well as in the regions of the Marmara sea and the Lake district (Özdoğan, Başgelen and Kuniholm 2012b, 2013). Based on details already described by several scholars, it can be stated that slightly differing Neolithic material cultures were in place and reflect fully-developed farming and herding societies in western Anatolia (e.g. Çilingiroğlu 2009; Galik and Horejs 2011; Lichter 2007; Özdoğan 2014; Duru 2008, pp. 119–121). Analysis of the centre of the Anatolian Aegean coast in this period of the Late Neolithic (c. 6500–5900/5800 BC) indicates closely-linked communities with quite homogeneous materiality (Horejs 2016). For Çukuriçi Höyük in this period (X–VIII), only those so-far unpublished aspects that shed new light on the relations of the Çukuriçi settlers to maritime networks will be presented here (for other categories see Horejs 2012, 2016).

**Fig. 11:**
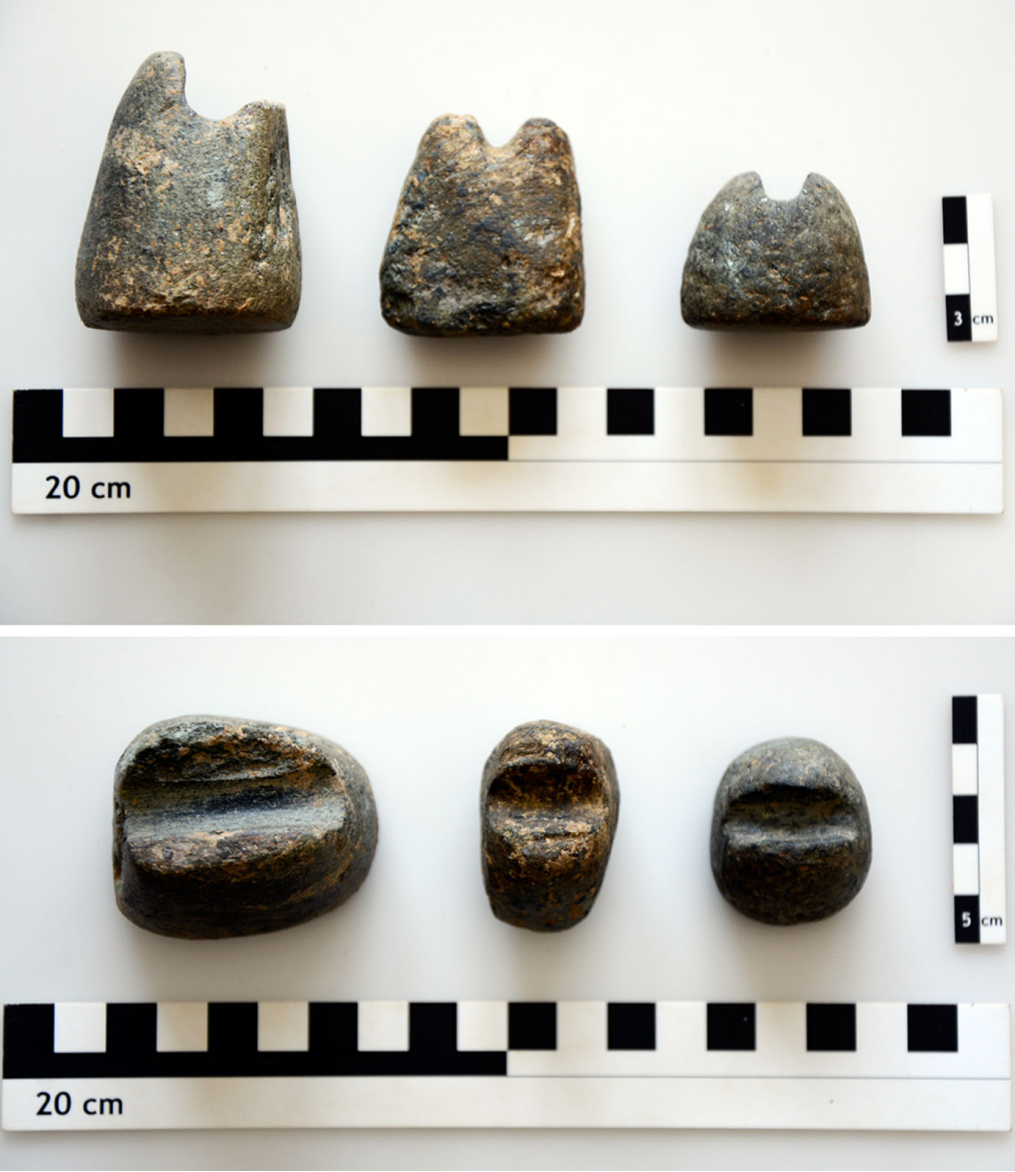
‘Horned shaft straighteners’ from Çukuriçi XI–X, with pedestal and flat bases (ERC Prehistoric Anatolia/F. Ostmann)

One exotic aspect of the material assemblages of Phases XI–X needs to be mentioned: Three shaft-straighteners with ‘horned pedestalled shape’ (Fig. 11) find their best parallels in PPN contexts of the southern Levant (Jordan valley and Black Desert), according to Kozlowski and Aurenche (2005, p. 159). This distribution might be less restricted if comparable horned pedestalled straighteners of, for example, Hallan Çemi (Rosenberg 2011), Körtik Tepe (Özkaya and Çoşkun 2011), Hasankeyf (Kozbe and Miyake 2014), or Demirköy (Rosenberg 2011) are included. Nevertheless, this specific type appears exotic in the Aegean and can again be related to the PPN core zone.

### Obsidian Procurement and Lithic Technology in the Late Neolithic

Once obsidian was introduced to Çukuriçi Höyük as an exotic, the settlement continued to procure the raw material from the island of Melos, resulting in the constant presence of obsidian in the lithic assemblages in extremely high amounts, up to 80%–86% in Phases X, IX and VIII. The presence of obsidian from Adamas and Dhemenegaki at Melos, attested by NAA, during the time span 6500–5900 calBC at Çukuriçi Höyük, shows a particularly long tradition of using the Aegean sources through the whole of the 7th millennium BC, while reliance on this source did not significantly change during the subsequent period of the occupation of the settlement in the Chalcolithic and Early Bronze Age (Bergner et al. 2008). Similarly, as raw material acquisition networks for obsidian and locally available cherts were stable through time, lithics from Phases X–VIII show continuity in technology, based on developed pressure debitage. Pressure-flaked blade making at Çukuriçi Höyük was carried out on conical and semi-conical cores, with complete core exhaustion resulting in bullet cores. The expertise in pressure flaking is documented through wide regular blades reaching sizes that can only be achieved by complex pressure techniques, using whole body mass, careful immobilization of the core, or special devices (modes of pressure technique after Pelegrin 2012). The analysis of blade dimensions and detachment stigmata demonstrated that sitting pressure with the core placed on the ground was the most widely-used mode for producing blades, while standing pressure was practised to a lesser extent. Blade fragments attesting significant widths, reaching more than 26 mm in obsidian and 20 mm in chert, could imply the existence of lever pressure, or at least experimentation in techniques. These new results in relation to technology demonstrate the high level of specialization among Çukuriçi artisans. So far, there is no clear evidence of specialized obsidian traders in the Aegean. Similarly, no particular site of the early to mid 7th millennium BC showing extremely high amounts of obsidian, with the developed local pressure technique, has been found in the eastern Aegean. Due to the fact that core preparation for pressure flaking plays a crucial role in the *chaîne opératoire* and must have been conducted by specialists, the procurement of obsidian directly from the source by settlement craftspeople can be suggested for the Neolithic community of Çukuriçi.

A tendency to make blade products on conical cores existed during the developed Neolithic in the second part of the 7th millennium BC in the Izmir region, while the major contrast lies in the proportion of obsidian among the lithic assemblages, which does not exceed 20% at the sites of Ulucak, Yeşilova and Ege Gübre (Milić 2014, p. 288, Fig. 2; Sağlamtimur 2011, p. 81). The common repertoire of tools can be observed within the micro region at all the sites mentioned, including Çukuriçi Höyük, consisting mostly of retouched blades, various kinds of end-scrapers, perforators, and sickle blades (Çilingiroğlu et al. 2012, p. 164; Derin 2011, p. 99; 2012, p. 182). Based on the published data on the technological features of Ulucak lithics, we can assume that flake blanks are much more frequent than blade blanks (Çilingiroğlu et al. 2012, p. 148), which indicates another important difference from Çukuriçi, where data sets are composed of masses of obsidian pressure-flaked blades, which might have been the result of raw material choice. The combination of exotic obsidian and technological knowledge makes Çukuriçi Höyük special. The location of the settlement, directly on the prehistoric coastline (Fig. 1), fits with the idea of its functioning as a gateway for raw material transport and distribution between the Aegean sea and sites in the western Anatolian interior.

**Fig. 12:**
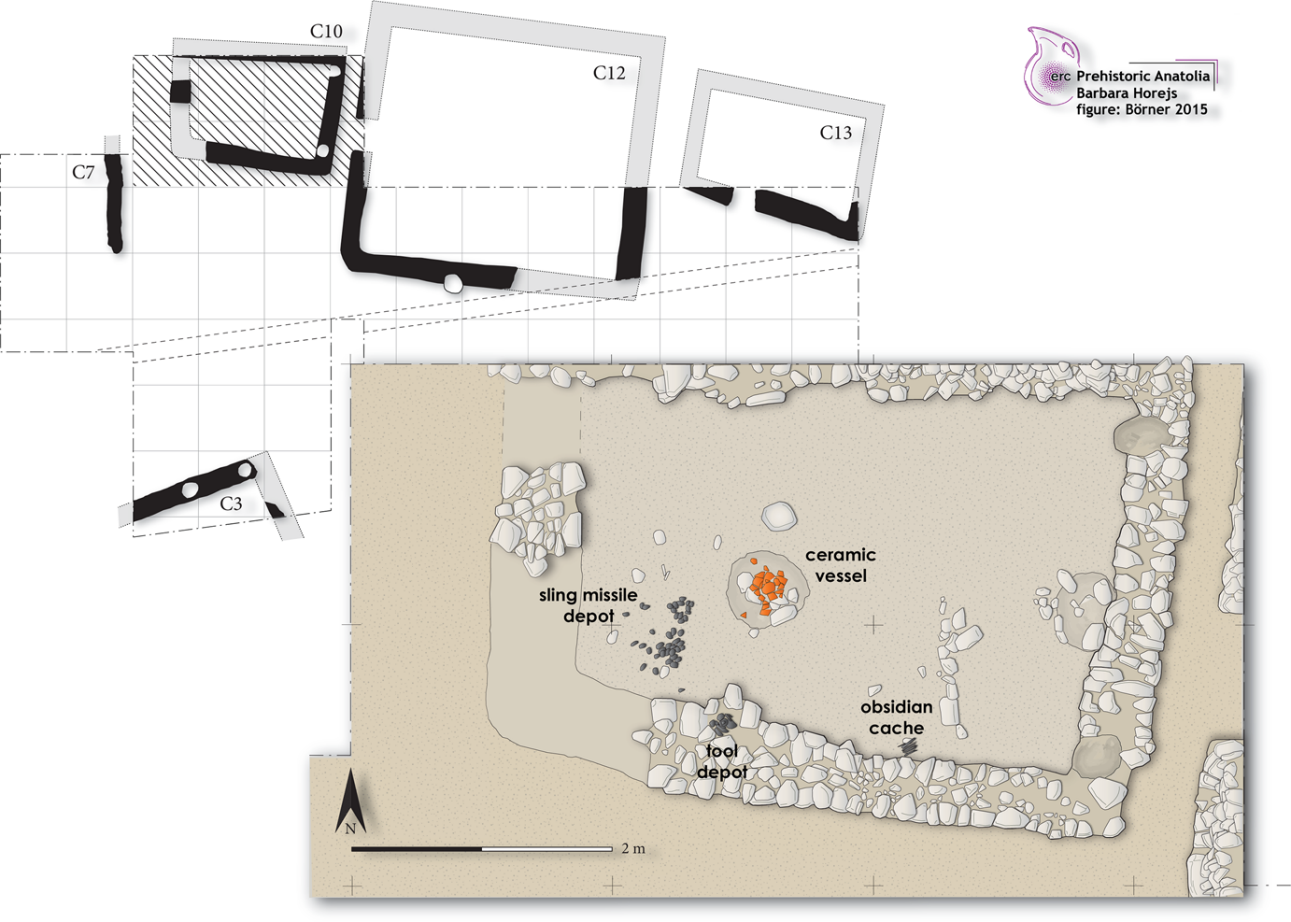
Archaeological remains of Phase X at Çukuriçi Höyük, with last preserved floor horizon of building Complex 10 as a blown-up detail of the cross-hatched area (ERC Prehistoric Anatolia/M. Börner)

Yet Çukuriçi Höyük’s special position in the Izmir region is due not only to the technological achievements manifest in ordinary lithic assemblages, but also to its remarkable artefact storage practices. The excavation of House Complex 10, within Phase X of Çukuriçi recovered different group finds in situ: a sling missile depot, a tool kit consisting of large retouched chert tools, and finally, a cache of long obsidian blades (Fig. 12). While depositing different tools together in certain places, such as house floors or pits, for future use seems to be common in contexts of developed Neolithic settlements over the larger area (e.g. deposits of sling missiles or lithic tools at Ulucak, Shir, Çatal Höyük: Çilingiroğlu et al. 2012, p. 145; Rokitta-Krumnow 2013, p. 233; Carter 2007), the cache of obsidian pressure blades from Çukuriçi is exceptional, with no current parallel in the Aegean prehistoric world.

### An Obsidian Cache and its Meaning for Interregional Connectivity

**Fig. 13:**
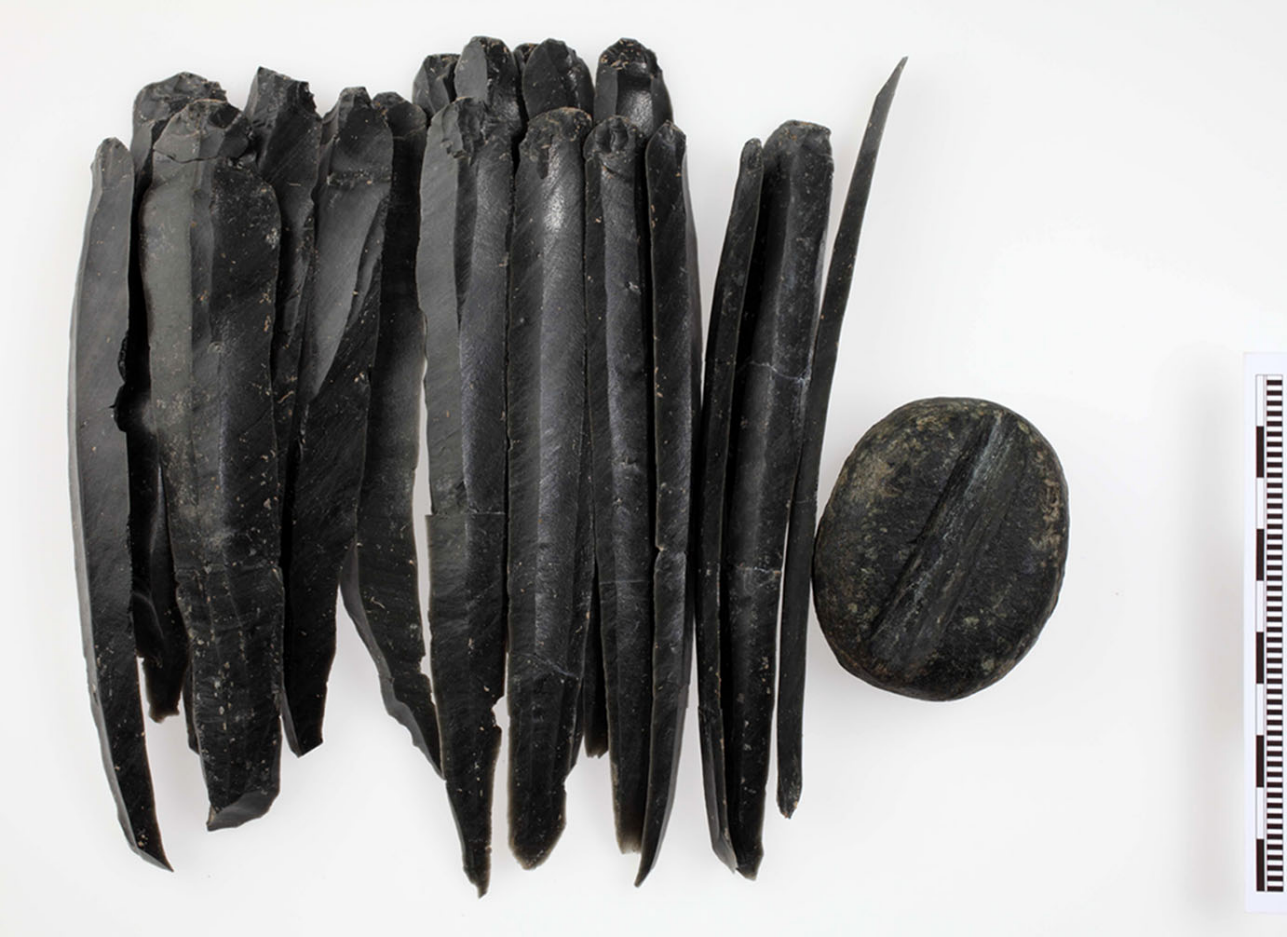
Obsidian cache with shaft straightener from Complex 10 of settlement Phase X at Çukuriçi Höyük (ERC Prehistoric Anatolia/N. Gail)

The cache of obsidian blades from Çukuriçi X consisted of 18 completely intact and unused long blades and was found inside Complex 10, in situ next to the house wall, in association with a single shaft straightener (Fig. 13). Only a few of the blades were broken in two, indicating post-depositional damage probably caused by sedimentation pressure. The longest blade measures 160 mm, with the average length for all blades being 147 mm. Technological examination shows that 13 of these blades were detached by application of a pressure-flaking technique, while the other 5 were probably obtained by indirect percussion. All blades belong to the category of central blades, and the absence of artefacts showing core preparation or containing cortex or natural surface implies the careful selection of objects for the cache. The perfect state of preservation and the close packing of the elements when found suggest that the group of blades had been bound with rope or string, and might additionally have been bundled in some kind of organic container.

The width range of the pressure blades in the cache is 15–22 mm, which is a function of standing pressure during detachment. Refitting could be performed in two sequences of three and four blades, and further examination on knapping rhythm may reveal whether they belonged to the same core. NAA showed that the blades originate from the same source within the Melian obsidian outcrops. In the same house, two further pairs of long obsidian blades were found on the floor and associated with the wall, but in different use horizons of the building. These blades were obtained by the same type of pressure technique, and are of similar length (110–143 mm), so may be considered to belong to the same core or cores as the long obsidian blades documented in the cache. Interestingly, those four blades were broken on distal parts, and two of them appear to be particularly slender, so it is still unclear whether they were excluded from the original cache or stored as small caches themselves.

As mentioned above, the same developed modes of pressure blade making at Çukuriçi pertained during Phases X–VIII among local artisans, but the long obsidian blades from the cache must still have represented a masterpiece of knapping. Since there was no preparation or production debris found in direct association with the cache or the other four long blades within House Complex 10, one may assume that knapping occurred elsewhere, within the settlement or in its direct vicinity. We would exclude the possibility that the blades were made at the obsidian source itself, mainly because of the perfect preservation of intact, fragile and slim blades. Secondly, the production of such blades would need the support of additional knapping kit, for example crutches or core-immobilization devices, which would have needed to be transported for each expedition to Melos. Therefore, we suggest that while the main material roughing out and shaping of nodules occurred at the source, Çukuriçi Höyük was where these long blades were knapped. Knapping on the spot is demonstrated by the presence of different cores and core preparation products, flakes and production debris in different contexts of the developed Neolithic, even though a specialized workshop has not yet been found.

Caches of long blades, mostly in flint, have been documented all over the Levant and in Upper Mesopotamia, with an isolated instance on Cyprus. They are connected exclusively with the Pre-Pottery Neolithic period, ranging from Early to Late PPNB. Numerous caches and stocks from the southern Levant have been mapped by Barzilai et al. (2007, p. 279, Table 1), while case studies have been published for other northern and southern Levantine sites: Motza (Khalaily et al. 2007); Yiftahel (Khalaily et al. 2013); ’Ain Ghazal (Karnes–Quintero 2007); and the Cypriot site of Shillourokambos (Briois 2007). All those deposits consisted of blades belonging to the bidirectional navifom industry, widespread among PPN lithic assemblages. There are only two contexts where PPNB caches of long blades detached by the pressure-flaking technique have been documented. The first is at the northern Syrian middle PPNB site of Sabi Abyad II, in the region of Upper Mesopotamia, a unique case in which long obsidian blades, detached by sitting or standing pressure, reach a maximum length of 13 cm (Astruc et al. 2007). At the second, Tell Ain el-Kerkh in northwestern Syria, two flint caches attest to the conjunction of bidirectional technology and pressure technique, and denote a lithic production trend in the northern Levant in the late PPNB (Arimura 2011). Finally, only two sites from the Pottery Neolithic period yielded information about caching or hoarding long blades after the PPNB: Çatalhöyük and Shir, in western Syria. Nevertheless, on both sites, long blades reveal a PPN tradition relying on bidirectional technology from the Levant (Carter 2007; Rokitta-Krumnow 2013).

The chronological context of caching from the Levant, Cyprus and Upper Mesopotamia demonstrated certain behaviours defined through the PPNB ‘cultural phenomenon’ (Barzai et al. 2007). Long blades were found in various contexts among the sites mentioned, in the inner space of houses, in fillings or sealed under plastered floors. Caches either consisted of unused long blades, or were in association with particular tools, mostly points and retouched blades, and it is suggested that they were often buried inside leather or textile bags, wooden boxes, or tied together with cords. According to the precise locations of finds and the *chaîne opératoire*, products stored together have been designated as deposits and caches, for functional or ritual (symbolic) use (Barzilai et al. 2007, p. 278; Astruc et al. 2003, p. 70).

An important fact pointed out by Carter in a case study of obsidian hoards from Çatalhöyük is that we do not always know whether the deposits we find belong to an intended first or last resting place after they have been made elsewhere (Carter 2007, p. 351). Two possible scenarios can apply to the cache of long obsidian blades from Çukuriçi Höyük. In the first, where the cache is deposited in the house immediately after production, we may have the temporary storage of blades prior to further circulation. In our opinion, this cache was not made with the intention of its future use within the settlement, because the Neolithic contexts were extremely rich in blade depositories. On the contrary, the concept of ‘keeping while giving’ (after Carter 2007, p. 352), which would involve a society possessing goods that could play a specific role in further trading or giving, due to their unique character, could be relevant in the case of the Çukuriçi cache. In considering the everyday functioning of Levantine sites, Barzilai et al. (2007) note the presence of stocks of high-quality artefacts within households in the large permanent villages of the eastern Mediterranean. This allows sites such as Motza, ’Ain Ghazal and Tell Abu Hureyra to be described as ‘distribution centers’ (Barzilai et al. 2007, p. 290). On the basis of the careful selection of long obsidian pressure blades showing craftsmanship in local production, and the remarkably high quantities of obsidian, the same model could be applied here for Çukuriçi Höyük. On the other hand, the two other groups of four long obsidian blades discussed above could be seen as deposits or reserves for future use by the settlers, since broken blades did not enter the cache. In the second scenario, if this was the intended final placement of an obsidian cache, one may think about special social behaviours in domestic contexts. Even though modern construction has damaged the uppermost layer of House Complex 10 where the cache was found, so that we do not have direct information proving that this was indeed the last use horizon, it seems that the cache was buried while the house was still in use, rather than made as part of any ritual or practice of abandonment, as documented from other areas of the site (Brami et al. 2016). However, bearing in mind that the storage of long blades is not common in the Aegean, and corresponds to specific PPN practices, the symbolic value of the cache should not be completely excluded, for instance in terms of burying items of special value as offerings made to building or to house-related communities respectively.

Comparisons between Çukuriçi and other PPNB caches from the above-mentioned sites imply close connections with finds from sites in northwestern and northern Syria, based on technological features and the character of unused long blades. Striking similarities can be seen with a parcel of bladelets from Sabi Abyad II, perfectly elaborated long, thin obsidian pressure blades, made by skilled knapper. It is important to note here that pressure-flaking debitage was absent from the local production at Sabi Abyad, while obsidian was imported from eastern Anatolia (Astruc et al. 2007, pp. 335–336); this constitutes the main difference from the Çukuriçi Höyük assemblages. Sabi Abyad appears to have represented a point where technological influence from the north, along with raw material procurement from that direction, fused with a Levantine tradition in symbolic representation to create a distinctive local community. On the other hand, the long obsidian blades from Çukuriçi do not show connections with caches from the Pottery Neolithic sites of Çatalhöyük and Shir, which somehow seem to be integrated in a pan-Levantine context through the tradition of making blades on bidirectional naviform cores. In conclusion, the caching of long blades at Çukuriçi Höyük on the western Anatolian coast presumably reflects practices visible in upper Mesopotamia during the mid 8th millennium BC, which were probably conserved for a thousand years through the principles of social memory and transmitting knowledge of past practice in the original core zone in some kind of *longue durée* process.

### Late Neolithic Subsistence Strategies

**Fig. 14:**
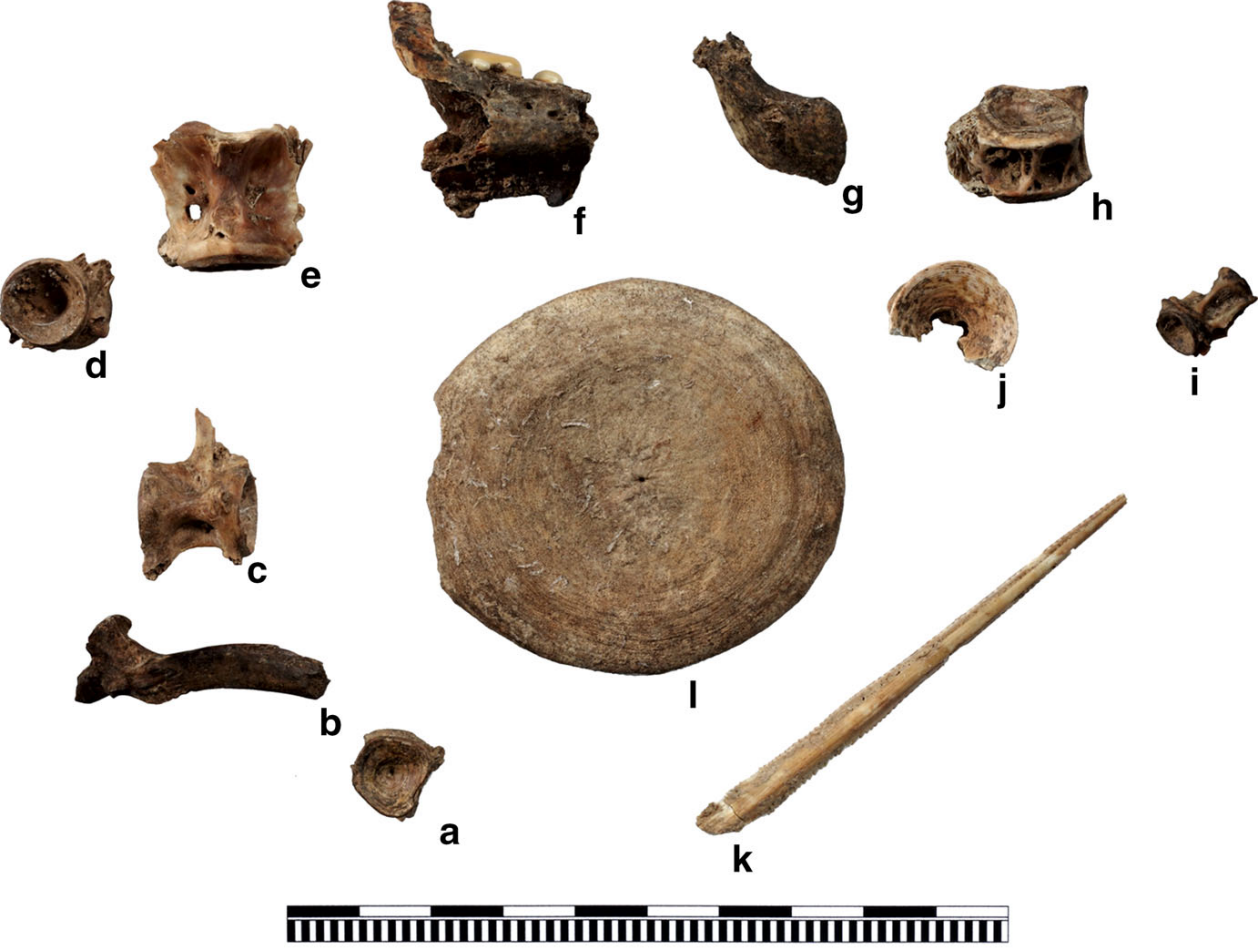
Fish- and whale-bones from Çukuriçi phases X–VIII; **a** thoracic vertebra Sparidae; **b** Maxilla Tuna; **c**–**e** caudal vertebrae Tuna; **f** dentale gilthead seabream; **g** maxilla gilthead seabream; **h** thoracic vertebra Sparidae; **i** caudal vertebra mackerel; **j** vertebra chondrichthes/shark; **k** sting of a stingray; **l** extremitas vertebralis caudalis dolphin/small whale (photo: ERC Prehistoric Anatolia/Niki Gail)

The subsistence patterns of the settlers at Çukuriçi Höyük in Phases X–VIII provide new insights into maritime affinities and networks, especially as the slightly differing, contemporaneous Neolithic material cultures reflect fully-developed farming and herding societies in western Anatolia. Phases X–VIII reveal continuity in subsistence strategies, with minor differences at Çukuriçi Höyük. The earlier phases indicate a moderate increase of marine animals towards Phase VIII, including remains of crustaceans and echinodermates (Fig. 9). The assemblages of domesticates are still dominated by ovicarpines and reveal a stable amount of cattle remains (Fig. 10). The frequency of identified domesticated pigs increased to reach 7% between Phase X and Phase VIII. Fallow deer remains the most important game animal, followed by hare, wild boar and fox (Table 3). In addition to small game such as wild cat, marten, and badger, a considerable amount of large game such as red deer, aurochs, roe deer, leopard and a bone from a small whale appear (Table 3). The low number of bird bones still argues for the minor importance of bird hunting even in the later phases. The composition of the ichthyofauna indicates the continued maritime affinity of the settlers. The littoral species like sparides, groupers and sea bass, as well as the pelagic tuna and mackerel and the cartilaginous fish, were still caught in Phases X–VIII. The composition of the molluscs did not significantly change during Phases X–VIII. The animal remains, especially with the bone of a small whale from Phase IX, confirm the continuity of the settlers’ maritime affinities towards 5900 cal BP (Fig. 14).

Much like the archaeozoological results, ongoing archaeobotanical investigation points to continuity in subsistence strategies in the Neolithic Phases X–VIII. Again, cereals and pulses are present throughout the site and barley appears to be the dominant crop. With the addition of naked wheat (*Triticum durum*/*aestivum*), the wheat assemblage became more diverse. Again, remains pertaining to crop processing at the site are missing. Towards the end of the sequence, flax or linseed (*Linum usitatissimum*) makes its first appearance.

## Discussion and Conclusion

The new excavation results from Çukuriçi Höyük at the centre of the Anatolian Aegean coastline are a starting point for proposing a revised model of neolithization for this region in the early 7th millennium BC. Viewed from the perspective of Anatolian and Levantine Neolithic trajectories, it has often been stated that the Aegean confronts us with a delayed initiation of neolithization. From a more global perspective, this pattern can also be seen in terms of differing Neolithic pathways, characterized by non-simultaneous trajectories and heterogeneous adaptation of constituent elements (Uchiyama et al. 2014).

**Fig. 15:**
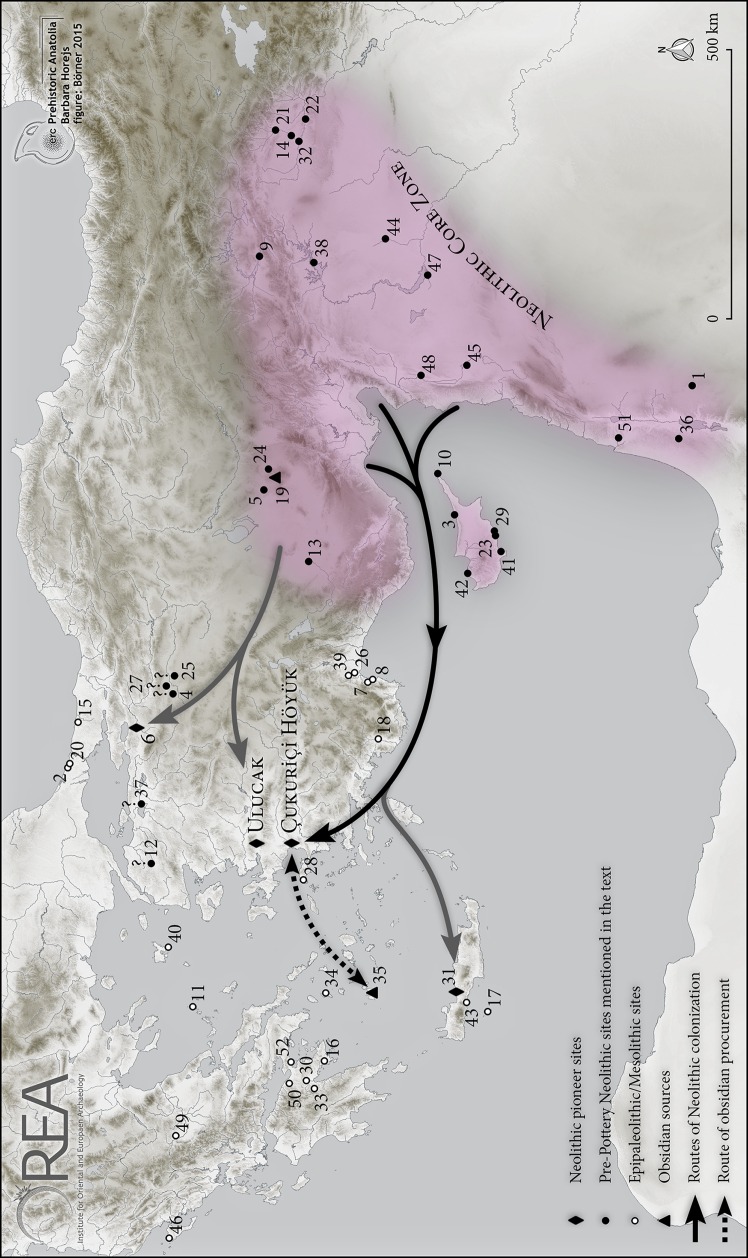
Neolithization of Western Anatolia with supposed routes of colonization (map: ERC Prehistoric Anatolia/M. Börner). *1* ’Ain Ghazal; *2* Ağaclı; *3* Akanthou/Tatlısu; *4* Asarkaya; *5* Aşikli Höyük; *6* Barcin Höyük; *7* Belbaşı; *8* Beldibi; *9* Cafer Höyük; *10* Cape Andreas Castros; *11* Cyclops Cave (Youra); *12* Çalca; *13* Çatal Höyük; *14* Demirköy; *15* Domalı; *16* Franchthi; *17* Gavdos; *18* Girmeler; *19* Göllü Dağ; *20* Gümüşdere; *21* Hallan Çemi; *22* Hasankeyf; *23* Kalavassos Tenta; *24* Kaletepe Kömürcü; *25* Kalkanlı; *26* Karain; *27* Keçiçayırı; *28* Kerame; *29* Khirokithia; *30* Klissoura; *31* Knossos; *32* Körtik Tepe; *33* Koukou; *34* Maroulas; *35* Melos; *36* Motza; *37* Musluçeşme; *38* Nevali Çori; *39* Öküzini; *40* Ouriakos; *41* Pareklisha Shillourokambos; *42* Petra tou Limnidi; *43* Plakias; *44* Sabi Abyad; *45* Shir; *46* Sidari; *47* Tell Abu Hureyra; *48* Tell Ain el-Kerkh; *49* Theopetra; *50* Ulbrich; *51* Yiftahel; *52* Zaimis

We are not able to fill the well-known gap between the Mesolithic evidence in the Aegean, northwest and southwest Anatolia and the first farmers and herders in the early Neolithic period. The new archaeological evidence from Çukuriçi Höyük supports the lack of a Pre-Pottery Neolithic period in the cultural sense in this region. Due to the current absence of patterns of transition from Mesolithic to Neolithic subsistence strategies at the centre of the Anatolian Aegean coast, we define Çukuriçi XIII and Ulucak VI as pioneer sites, founded by newcomers around 6700 cal BC (Fig. 15). Barcin Höyük in the Marmara region also appears to be a pioneer site, as indicated by the recently published radiocarbon sequence (Weninger et al. 2014, p. 21, Fig. 14). The founding horizon of Çukuriçi demonstrates a community that relied on farming and herding but also on shell-fishing and fishing. This particular set of technologies, skills like seafaring and a combined subsistence strategy, seem most probably to have been brought to this site by the newcomers. The ichthyofaunal composition of littoral and pelagic species at the earliest Phases XIII–XI of Çukuriçi Höyük resembles the exploitation pattern demonstrated at early sites in the Mediterranean (Bar-Yosef Mayer 2014). Phases XIII–XI from Çukuriçi Höyük reveal a noticeable increase of large bones, in the form of wild animals amongst the bovid, ovicaprid and suid remains. On the one hand, despite their relative paucity, these probably indicate the decline of animals with an ancestral morphology. On the other hand, the significant number of clearly identifiable domesticates undoubtedly reveals exploitation of domesticated and herded flocks in the foundation phase of Çukuriçi Höyük.

We conclude that the pioneer sites on the central Aegean coast of western Anatolia most likely represent agricultural enclaves within ‘empty landscapes’. It has been argued that the absence of Mesolithic groups (i.e. precisely the fact that this area was not occupied by other populations) might have been an important impetus for the newcomers’ choice. This model, also discussed for other regions in the wider Mediterranean, might explain the absence of a transformation-and-adaptation process from hunter-gatherers to early farmers (cf. Pearce 2013).

Integrating our outcome into the broader complexity of Mediterranean Neolithic pathways (Perlès 2001; Broodbank 2006), comparable patterns are recognizable elsewhere. The ‘maritime pioneer colonization model’ (Zilhão 2001), put forward as the main trigger for the neolithization of the Iberian Peninsula, shows a similar pattern of early farming enclaves, potentially founded by leapfrogging seafaring colonists. Indisputably, the neolithization of the Mediterranean coastal zones was profoundly affected by mobile groups and seafaring connectivity (Broodbank 2006).

We have argued for maritime routes of colonization for the eastern Aegean seaboard villages, and Çukuriçi Höyük in particular, rather than terrestrial migration from inland Anatolia. Although the question of the colonizers’ origin must remain open, a relation to the Neolithic core zone, and the especially the Levant, has been discussed, based on technology, material and material practices. The material assemblage of the Çukuriçi founding period indicates a relationship to the Neolithic core zone, especially to the later PPN period of the 8th millennium BC (as discussed above). The majority of characteristic PPNB elements in the Levant and upper Mesopotamia are certainly absent at Çukuriçi XIII, which was founded some centuries later. However, the malachite bead, the horned shaft-straighteners and the stone bracelet all have their only and best parallels in the core zone. Even though movements from the Levantine corridor to regions far away (Ouriakos on Lemnos) from the core zone are already proposed for the Epipalaeolithic period (Efstratiou et al. 2014), in our view more data are needed to confirm such activities before the early Neolithic. However, the particular connections of the Çukuriçi newcomers to the core zone may be related to the maritime networks in the Mediterranean (Fig. 15).

It has been pointed out that in the pre-Neolithic sequences only regional networks, relating to the procurement of Melian obsidian, existed in the Aegean. No complex system with exchange and distribution at larger scales (as in the case of Cappadocian obsidian dispersal that extended all the way down to the Levant and to Cyprus), was ascribed to the early communities of the Aegean before the 7th millennium BC. This has been interpreted as the result of basic knowledge limitations concerning the specialized lithic technologies needed for the production of standardized tools. Such tools could have been a trigger for obsidian demand over broader regions in the Aegean. Groups with seafaring knowledge were on the scene early, and the high quality obsidian outcrops at Melos would certainly have been the preferred exotic raw material. The procurement of obsidian at the onset of the Neolithic on both sides of the Aegean is documented from the earliest levels of Neolithic settlements, including at Knossos, Franchthi, Argissa (Conolly 2008) and Çukuriçi Höyük in the first half of the 7th millennium BC. However, obsidian is represented differently in each setting due to differences in maritime connectivity, cultural background, local habitat and external influences.

The arrival of the newcomers in the Aegean can be chronologically associated with the introduction of a new lithic technology based on the pressure-flaking technique. In combination with particular chipped stone tool types and the presence or absence of pre-Neolithic occupation, different processes of neolithization in Crete, Argolid and Thessaly have been suggested (Conolly 2008, p. 85; Perlès 2001). However, the initial spread of pressure-flaking techniques from the core zone and their full development to the levels of mastery seen at Çukuriçi Höyük distinguish that site from contemporaneous settlements in the wider region. Lithic assemblages from fully developed Neolithic western Anatolia (after c. 6500 BC) illustrate intense Melian obsidian procurement, pointing to the coastal site of Çukuriçi as a focal point receiving extremely high amounts of obsidian. The already debated model for obsidian distribution on the northern coast of Cyprus in the 9th millennium BC (Şevketoğlu and Hanson 2015) could be applied to Cukurici Höyük’s obsidian procurement at Melos in relation to western Anatolian sites in the 7th millennium BC. The network whose existence we propose was, possibly a uniquely important major gateway in early Aegean prehistory, predicated on the existence of a specialized technology of exogenous origin.

Recent ancient DNA studies of human remains in Near East, neighbouring regions and Europe support a maritime route for European colonization in the Neolithic (Haak et al. 2010; Paschou et al. 2014), including Cyprus and the Aegean islands (Fernanández et al. 2014). These human aDNA studies track the same maritime distribution pattern revealed by analyses of a huge data set of zoological patterns recently published by Arbuckle et al. (2014, Fig. 1).

We propose a maritime colonization in the 7th millennium BC, via routes from the eastern Mediterranean to the eastern Aegean based on previously established marine connections (Fig. 15). The newcomers arrived with their package of nautical know-how, most probably linked to eastern Mediterranean seaborne networks. Their knowledge of routes, navigation, sources and all aspects of successful seafaring appears to have been used by groups in the early 7th millennium BC in exploring the centre of the Anatolian Aegean coast to establish some of the first permanent settlements in the region. The archaeological evidence of Çukuriçi Höyük can most probably be related to maritime colonization rather than migrations from inland Anatolia. These farming and herding societies let us observe traces of earlier PPN concepts of materiality that remained embedded in the social-cultural memories of the newcomers (Assman 1992; Özdoğan 2014, p. 84) and which were incorporated in a new local and regional Neolithic identity developed within a very few generations. As little as 200 years later, at c. 6500 BC, the centre of the Anatolian Aegean coast shows a particular regional connectivity, which probably relates to the first emergence of this distinctive regional Neolithic identity.
